# Harnessing the gut–immune–joint axis: Oral microalgae-based thermoresponsive microspheres enhance intra-articular therapy for rheumatoid arthritis

**DOI:** 10.1016/j.bioactmat.2026.01.037

**Published:** 2026-02-10

**Authors:** Ruoxi Wang, Aiying Tong, Kangyu Jin, Runchang Yu, Donghu Lin, Di Yang, Xiaoyang Liu, Jiarong Cui, Jiahua Niu, Yulin Cui, Haishuang Zhu, Min Zhou

**Affiliations:** a Eye Center, The Second Affiliated Hospital, Zhejiang University School of Medicine, Hangzhou, 310000, China; b Institute of Translational Medicine, Zhejiang University, Hangzhou, 310029, China; c Zhejiang University-University of Edinburgh Institute (ZJU-UoE Institute), Zhejiang University School of Medicine, Zhejiang University, Haining, 314400, China; d Zhejiang University-Ordos City Etuoke Banner Joint Research Center, Haining, 314400, China; e School of Pharmacy, Binzhou Medical University, Yantai, 264003, China

**Keywords:** Rheumatoid arthritis, Intestinal barrier, *Chlorella vulgaris*, Ginseng polysaccharides, Gut microbiota modulation

## Abstract

Rheumatoid arthritis (RA) is a chronic autoimmune disease primarily caused by an aberrant immune response that erroneously attacks the synovial joints, leading to inflammation and joint damage. Emerging evidence suggests that impaired intestinal barrier integrity and imbalanced gut microbiota play crucial roles in driving RA development, promoting systemic inflammation, and exacerbating joint pathology. Here we propose a synergistic therapeutic strategy that concurrently addresses both the systemic gut-immune axis and local joint inflammation. This approach integrates intra-articular injection of triamcinolone acetonide (TAA) with oral administration of thermoresponsive microspheres encapsulating Chlorella vulgaris (CV) and ginseng polysaccharides (GPS), designated as CG@GelMA. The microspheres undergo temperature-induced gelation at body temperature, thereby facilitating gastric transit and enabling prolonged drug release in the intestinal tract. Oral administration of CG@GelMA restored intestinal barrier function by enhancing tight junction protein expression and exerting anti-inflammatory effects, while intra-articular TAA synergistically alleviated synovial inflammation, improved locomotor function, and preserved bone and cartilage integrity. Moreover, the combination therapy elicited superior immune modulation, characterized by increased regulatory T cells, reduced Th17 cells, and a systemic cytokine shift toward elevated interleukin-10 and reduced interleukin-17. Notably, this systemic immunomodulation was driven by CG@GelMA-mediated remodeling of the gut ecosystem, which enriched beneficial taxa (e.g., *Lactobacillus*), reduced potentially pathogenic genera (e.g., *Escherichia–Shigella*), and, importantly, led to a significant increase in the intestinal levels of immunomodulatory metabolites, including several short-chain fatty acids (SCFAs). Fecal microbiota transplantation (FMT) and depletion studies definitively established the gut microbiota as the central mediator of these therapeutic effects. Together, these findings highlight a synergistic combinatorial strategy that couples microbiota-driven systemic immunomodulation with potent local anti-inflammatory effects, offering a promising avenue for the treatment of RA and other systemic inflammatory disorders.

## Highlights

• Thermoresponsive microspheres (CV/GPS@GelMA) deliver *Chlorella vulgaris* and ginseng polysaccharides to intestine for RA.
• The system boosts intestinal barrier function via tight junction upregulation, reduced oxidative stress and gut inflammation.
• With intra-articular triamcinolone acetonide, it eases joint inflammation, preserves bone and boosts mobility in RA mice.
• Gut microbiota remodeling (increased *Ligilactobacillus*, decreased pathogens) mediates systemic immunomodulatory effects.
• Fecal microbiota transplantation verifies gut microbiota’s key role in the combined treatment’s therapeutic effects.

## Graphical abstract

**Figure:**
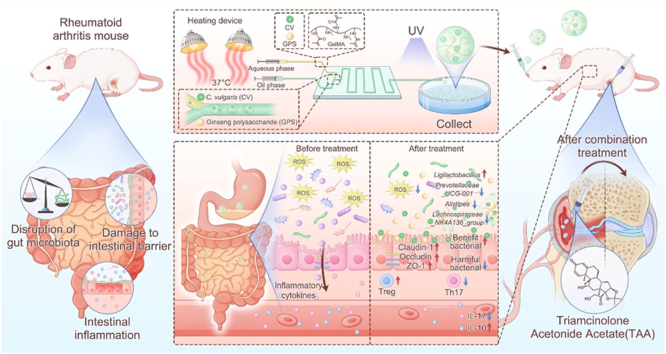

## 1 Introduction

Rheumatoid arthritis (RA) is a chronic autoimmune disease affecting approximately 1 % of the global population and remains a major contributor to disability and healthcare burden worldwide [1,2]. Beyond the classical view of RA as a joint-centric inflammatory condition, accumulating evidence supports its characterization as a systemic disorder involving complex immune dysregulation [[3], [4], [5], [6]]. However, current clinical paradigms remain largely centered on either local anti-inflammatory therapy at the joint level or systemic immunosuppression, which often carries significant off-target effects [7,8]. Recent studies increasingly focus on the role of the intestinal epithelial barrier in systemic immune regulation and its potential involvement in RA pathogenesis [9,10]. Disruption of the intestinal epithelial barrier allows harmful microbial metabolites and pro-inflammatory mediators to enter the systemic circulation, thereby promoting innate immune activation and systemic inflammation [[11], [12], [13], [14]]. This inflammatory state can impair peripheral immune tolerance and disrupt the balance of immune cells, such as regulatory T cells (Tregs) and pro-inflammatory Th17 cells, whose dysregulation has been implicated in various autoimmune diseases, including RA [[15], [16], [17], [18]]. Emerging evidence suggests that restoring mucosal barrier integrity—through dietary interventions, microbiota modulation, or pharmacological treatments—can reduce inflammation and immune imbalance, highlighting the intestinal barrier as a promising therapeutic target [[19], [20], [21], [22], [23]].

The gut microbiota is recognized as a key factor in maintaining intestinal barrier function and immune homeostasis [[24], [25], [26]]. Studies have shown that microbial dysbiosis occurs in both RA patients and animal models, suggesting a potential role in disease development [[27], [28], [29], [30]]. Notably, several bacterial taxa associated with impaired mucosal integrity and inflammation have been implicated in RA pathogenesis. For instance, *Prevotellaceae_UCG-001* [[31], [32], [33]], *Alistipes* [34], and *Lachnospiraceae_NK4A136_group* [35,36] have been linked to increased intestinal permeability and pro-inflammatory responses. In contrast, beneficial commensals such as Ligilactobacillus are associated with improved epithelial barrier function and modulation of immune-metabolic pathways [[37], [38], [39], [40]]. These microbial shifts can influence the production of key metabolites, particularly short-chain fatty acids (SCFAs) [41,42], which help stabilize the mucus layer, upregulate tight junction proteins, and modulate immune responses [[43], [44], [45]]. Thus, the depletion of beneficial taxa (e.g., *Ligilactobacillus*) alongside the expansion of permeability-associated taxa (e.g., *Prevotellaceae_UCG-001*, *Alistipes*) collectively contributes to a “leaky gut” state, fueling systemic inflammation in RA [30,[46], [47], [48], [49]]. Therefore, targeted modulation of the gut microbiota may represent a promising approach to restore epithelial and immune balance in RA.

Given the central role of intestinal barrier dysfunction and gut microbiota dysbiosis in RA pathogenesis, strategies to restore barrier integrity and microbial balance are gaining attention. Natural bioactive compounds stand out as promising candidates due to their safety and multifaceted regulation of gut–immune homeostasis [[50], [51], [52]]. Ginseng polysaccharide (GPS), a bioactive fraction derived from ginseng, has demonstrated protective effects on the gut and immunomodulatory functions in multiple studies [[53], [54], [55]]. GPS enhances epithelial barrier function by promoting mucin production and upregulating tight junction proteins including Claudin-1, Occludin and ZO-1, while simultaneously bolstering intestinal immune defense by enhancing secretory IgA production and reinforcing the physicochemical barrier through upregulation of antimicrobial peptides and E-cadherin [56,57].

Building on this, the nutrient-rich unicellular alga *Chlorella vulgaris* (CV) exhibits gut- and immune-regulatory effects that complement those of GPS [[58], [59], [60]]. Owing to its rich composition of bioactive constituents such as polysaccharides and peptides, CV modulates immune responses and gut homeostasis through multiple mechanisms, including direct interaction with host pathways and serving as substrates for the production of microbiota-derived metabolites such as SCFAs. Accordingly, CV has been shown to attenuate Th17-mediated inflammation and modulate cytokine responses, thereby enhancing mucosal immune tolerance [58,61]. Notably, CV directly protects the intestinal epithelium through its anti-inflammatory and antioxidant effects [62]. Therefore, our approach capitalizes on this synergistic interplay: GPS primarily acts to restore and reinforce the gut barrier and immune tolerance, while CV provides crucial protection by mitigating the inflammatory and oxidative insults that challenge epithelial integrity. Together, they form a multi-targeted strategy that concurrently addresses microbial dysbiosis, barrier defects, and immune dysregulation in RA.

However, oral delivery of water-soluble bioactive compounds such as GPS and CV faces challenges due to gastrointestinal degradation, poor mucosal adherence, and low intestinal bioavailability. To address these limitations, we developed a delivery platform based on methacryloyl gelatin (GelMA), a thermoresponsive hydrogel derived from collagen. GelMA undergoes a sol–gel transition near physiological temperature and is stable under acidic conditions [63]. This enables the microspheres to protect the encapsulated bioactives under gastric conditions and allow controlled release in the intestine. The hydrogel network of GelMA ensures high loading efficiency, while its crosslinking properties allow fine-tuning of mechanical strength, swelling behavior, and drug release kinetics. Furthermore, the intrinsic strong red autofluorescence of CV provides a built-in optical tracer, enabling direct, non-invasive visualization and real-time monitoring of the microspheres' distribution, retention, and targeting efficiency within the gastrointestinal tract, which is crucial for evaluating the performance of the oral delivery system.

**Scheme 1:**
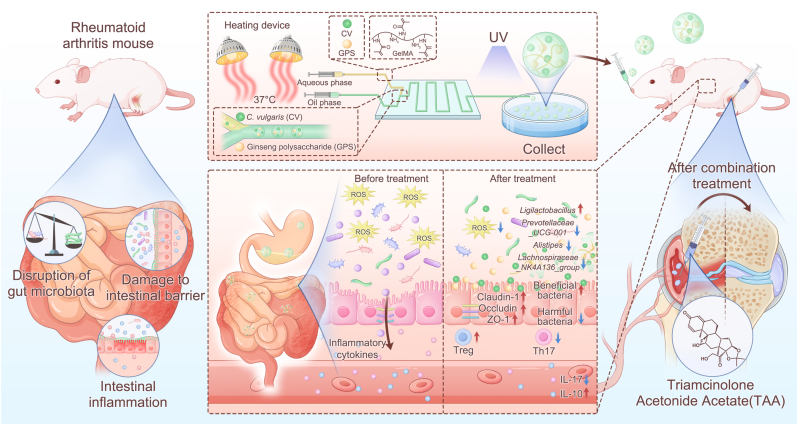
Schematic illustration of a combinatorial therapeutic strategy for rheumatoid arthritis (RA) involving intra-articular injection of triamcinolone acetonide (TAA) and oral administration of thermoresponsive CG@GelMA microspheres. These GelMA-based microspheres, co-loaded with *Chlorella vulgaris* (CV) and ginseng polysaccharide (GPS), are fabricated via a microfluidic platform. Upon oral delivery, CG@GelMA resists gastric degradation and enables sustained intestinal release, where it restores gut homeostasis by upregulating tight junction proteins, reducing oxidative stress and inflammation, and reshaping the gut microbiota—enriching SCFA-producing bacteria while suppressing pro-inflammatory taxa. These changes enhance gut barrier integrity and alleviate intestinal inflammation. Systemically, the treatment promotes an anti-inflammatory immune shift, characterized by increased splenic Treg cells and reduced Th17 cells, followed by elevated serum IL-10 and decreased IL-17 levels. In combination with local TAA injection, this dual-targeted approach effectively relieves both joint inflammation and intestinal dysfunction, offering a synergistic RA treatment strategy via modulation of the gut–joint axis.

In this study, we constructed GelMA-based microspheres co-encapsulating GPS and CV (CG@GelMA) to achieve gastric acid resistance, controlled release, and prolonged retention in the intestinal tract. In a collagen-induced arthritis (CIA) mouse model, oral administration of CG@GelMA improved gut barrier integrity, as evidenced by reduced oxidative stress, upregulated tight junction proteins (Claudin-1, Occludin, and ZO-1), and attenuated systemic inflammation. These intestinal improvements were accompanied by immunological modulation, including reduced Th17 cell frequencies and IL-17A expression and increased Treg frequencies and IL-10 levels. This immune shift helped prevent excessive intestinal leakage and mitigated subsequent joint inflammation. Combining oral CG@GelMA with intra-articular triamcinolone acetonide (TAA) further amplified joint-level therapeutic effects. Fecal microbiota transplantation (FMT) revealed that the gut microbiota was essential for this response: transplantation from CG@GelMA - treated donors alleviated joint inflammation, synovial hyperplasia, and bone erosion, whereas concurrent antibiotic treatment abolished these benefits. Together, these findings demonstrate a safe and effective adjunctive RA therapy combining two bioactive food-derived agents delivered via a controlled-release microsphere system. CV and GPS synergistically restore intestinal barrier integrity, modulate the gut microbiota, and rebalance systemic immunity, while co-administration with intra-articular treatment enhances control over RA progression. Herein, we present a novel therapeutic paradigm that integrates intra-articular intervention with an oral therapy designed for concurrent restoration of the gut barrier and microbiota, thereby establishing a synergistic interplay between local symptom control and systemic immunomodulation (Scheme 1).

## 2 Materials and methods

### 2.1 Fabrication and characterization of thermoresponsive CG@GelMA

CG@GelMA were synthesized using a microfluidic system (ELF-MS-1000, Engineering for Life, Jiangsu, China) to ensure uniform particle morphology. Briefly, 12.5 mg of GelMA was fully dissolved in 5 mL of PBS containing 12.5 mg of the photoinitiator LAP. *Chlorella vulgaris* (CV) cells (Guangyu Biological Technology, Shanghai, China) were harvested by centrifugation at 4500 rpm for 10 min and rinsed three times with PBS. Subsequently, 7 × 10^7^ CV cells and 10 mg of ginseng polysaccharide (GPS; Shanghai Macklin Biochemical Co., Ltd) were suspended in the GelMA-LAP solution, serving as the aqueous dispersed phase. The oil phase was prepared by mixing 5 mL Span 80 with 45 mL paraffin oil and preheating it at 40 °C for 10 min. Both phases were delivered into the microfluidic chip via precision pumps, where shear force at a water-to-oil flow ratio of 4:32 generated monodisperse droplets. Throughout the process, the system was maintained at 37 °C using a heating lamp to preserve the low viscosity of GelMA. The generated droplets were collected and immediately crosslinked via UV irradiation (365 nm, 80 mW/cm^2^), forming structurally stable microspheres. The final products were washed thoroughly with PBS to remove residual oil. The morphology of the microspheres was examined using scanning electron microscopy (SEM; SU-70, Hitachi, Japan). Optical and fluorescence microscopy (Zeiss, Germany) were used to visualize microsphere structure and CV autofluorescence. UV–visible absorption spectra were recorded with a UV-2600 spectrophotometer (Shimadzu, Japan), and fluorescence emission spectra were obtained using an RF-6000 spectrofluorometer (Shimadzu, Japan) at an excitation wavelength of 552 nm. Zeta potential analysis was carried out using a Nano-ZS90 instrument (Malvern Instruments, UK) to assess surface charge characteristics. Structural composition and functional group distribution were confirmed via Fourier-transform infrared spectroscopy (FTIR; Shimadzu, Japan) across a scan range of 500–4000 cm^−1^.

### 2.2 Cell culture and in vitro intestinal barrier model

RAW 264.7 macrophages (Procell, Wuhan, China), Caco-2 intestinal epithelial cells (Pricella, Wuhan, China), and IEC-6 small intestinal epithelial cells (ATCC, USA) were maintained in high-glucose DMEM supplemented with 10 % fetal bovine serum (AiTing, Hangzhou, China) and 1 % penicillin-streptomycin (Gibco, USA), with the medium for IEC-6 cells additionally containing 0.1 U/mL human insulin. Cells were cultured at 37 °C in a humidified incubator containing 5 % CO_2_. To mimic RA-associated gut dysbiosis, fecal supernatants (FSN) were prepared by homogenizing stool samples from CIA mice in sterile PBS to a 10 % (w/v) suspension, followed by centrifugation (4000×*g*, 10 min) and filtration through 1.2 μm membranes. Supernatants were aliquoted and stored at −80 °C. For *in vitro* co-culture experiments, Caco-2 cells were seeded on Transwell inserts (Corning, USA) and differentiated over 14 days to establish polarized monolayers. RAW 264.7 macrophages were seeded in the basolateral chamber 3 days prior to treatment. To simulate inflammatory challenge, FSN was added to the basolateral compartment. For intervention, CV (1.4∗10^5^ cells/mL), GPS (20 μg/mL), Blank@GelMA (25 μg/mL), or CG@GelMA (CV = 1.4∗10^5^ cells/mL, GPS = 20 μg/mL, Blank@GelMA = 25 μg/mL) were applied to RAW 264.7 1 h before FSN exposure.

### 2.3 Assessment of barrier integrity and molecular analysis

Transepithelial electrical resistance (TEER) was measured using a Millicell ERS-2 volt-ohm meter (Millipore, USA) at baseline and every 2 h for 12 h. The theoretical additive TEER value was computed using the following formula:

$$\text{Theoretical}\;\text{TEER}={\text{TEER}}_{\text{FSN}}+\left({\text{TEER}}_{\text{CV}}-{\text{TEER}}_{\text{FSN}}\right)+\left({\text{TEER}}_{\text{GPS}}-{\text{TEER}}_{\text{FSN}}\right)\text{.}$$

Values were averaged from three independent positions per insert. To assess paracellular permeability, fluorescein isothiocyanate-dextran (FD4, 4 kDa; Sigma-Aldrich) was added to the apical side at 1 mg/mL. After 2 h, basolateral samples were collected and fluorescence intensity was recorded (Ex/Em = 492/520 nm) using a microplate reader (SpectraMax iD5, Molecular Devices, USA). For tight junction (TJ) protein detection, Caco-2 monolayers were fixed and stained with antibodies against Claudin-1 (Abnova, China), Occludin (Abcam, UK), and ZO-1 (Abcam, UK), followed by DAPI (Servicebio, Wuhan, China) counterstaining. Fluorescence images were captured using a laser scanning confocal microscope and analyzed with ImageJ software.

### 2.4 Oxidative stress and cell viability assays

To evaluate intracellular oxidative stress, IEC-6 cells were seeded into 96-well plates and stimulated with LPS (25 μg/mL), alone or with CV (1.4∗10^5^ cells/mL), GPS (20 μg/mL), Blank@GelMA (25 μg/mL), or CG@GelMA (CV = 1.4∗10^5^ cells/mL, GPS = 20 μg/mL, Blank@GelMA = 25 μg/mL) for 12 h. After treatment, cells were co-stained with Hoechst 33342 and DCFH-DA (YEASEN, Shanghai, China). ROS levels were visualized using a fluorescence microscope. Cell viability was assessed using Calcein-AM/PI (Solarbio, Beijing, China) dual staining and CCK-8 assays (YEASEN, Shanghai, China). After 12-h co-incubation under LPS challenge, IEC-6 cells were stained with Calcein-AM and PI to distinguish live (green) and dead (red) cells. Separately, CCK-8 solution was added to treated cells for 2 h, and absorbance at 450 nm was measured with a plate reader.

### 2.5 Evaluation of in vitro antioxidant activity

The scavenging capacities against superoxide anion (O_2_·^-^), hydrogen peroxide (H_2_O_2_), and hydroxyl radical (·OH) were assessed for CV, GPS, CV + GPS, Blank@GelMA and CG@GelMA. Superoxide anion (O_2_·^-^) scavenging assay: The scavenging capacity was evaluated using the nitroblue tetrazolium (NBT) method. Briefly, samples at varying concentrations were mixed with a reaction solution containing riboflavin (20 μM), methionine (12.5 mM), and NBT (75 μM). The mixture was then exposed to ultraviolet light for 15 min, and the absorbance was measured at 560 nm. Hydrogen peroxide (H_2_O_2_) scavenging assay: The scavenging activity was determined using a TMB-based method. Samples were incubated with a commercial H_2_O_2_/TMB solution for 10 min, and the absorbance was recorded at 405 nm. Hydroxyl radical (·OH) scavenging assay: The scavenging capacity was measured using the salicylic acid (SA) method. Hydroxyl radicals were generated via the Fenton reaction. Samples were added to the reaction system containing SA, and the absorbance of the resulting 2,3-dihydroxybenzoic acid was measured at 510 nm. The inhibition rate was calculated as: Inhibition rate (%) = [A_0_ - (A_x_ - A_x0_)]/A_0_ × 100 %, where A_0_ is the absorbance of the blank control, A_x_ is the absorbance of the sample, and A_x0_ is the absorbance of the sample background without H_2_O_2_.

### 2.6 In vivo gastrointestinal tracking and biodistribution analysis

All procedures involving animals were conducted in accordance with protocols approved by the Institutional Animal Care and Use Committee of Zhejiang University (approval number: ZJU20250848). To investigate the gastrointestinal transit and biodistribution profile of CG@GelMA, female Balb/c nude mice (6 weeks old; n = 3 per time point) were utilized for real-time *in vivo* fluorescence imaging. Mice were fasted overnight prior to oral gavage with 300 μL of one of the following formulations: CV, FITC, or CV/FITC@GelMA. The concentrations of CV and FITC in the dosing solutions were 1.4 × 10^7^ cells/mL and 2 mg/mL, respectively. Fluorescent signals were captured at predefined time points (0.5, 1, 2, 3, 4, 6, 8, 10, 12, 24, and 48 h) post-administration using the IVIS Lumina LT Series III imaging system (PerkinElmer, USA), under isoflurane anesthesia. Quantitative analysis was performed using Living Image 4.5 software. At select time intervals, mice were sacrificed to collect major organs (heart, liver, spleen, lungs, kidneys) and segments of the gastrointestinal tract (stomach, intestine, cecum, and colon) for *ex vivo* fluorescence imaging. To further characterize microsphere transit and degradation, luminal contents from different intestinal segments (stomach, ileum, cecum, colon) were collected following gavage with CV/FITC@GelMA and observed using scanning electron microscopy (SEM). Additionally, fecal samples were collected at intervals (0.5–8 h post-gavage) and imaged under the CV fluorescence channel to monitor excretion kinetics and gastrointestinal clearance of the microspheres.

### 2.7 Identification of arthritis-sensitive and resistant phenotypes

To induce collagen-induced arthritis (CIA), male DBA/1 mice (6–8 weeks old) were intradermally immunized at the tail base with 100 μg of bovine type II collagen emulsified in Complete Freund's Adjuvant (CFA) on day 0, followed by a secondary immunization using collagen in Incomplete Freund's Adjuvant (IFA) on day 21. On day 25, based on clinical observations, mice were retrospectively categorized into sensitive group and resistant group. Sensitive group displayed prominent joint inflammation and achieved clinical scores ≥4 in at least one paw, whereas resistant group showed no visible arthritis symptoms (score = 0). Systemic status was monitored through body weight and spleen size assessments. At days 20 and 50, intestinal permeability was evaluated by serum FITC–dextran (administered one day prior) and zonulin (measured at sacrifice). Intestinal tissues were collected for histopathological staining and immunofluorescence imaging of tight junction proteins, including Claudin-1, Occludin, and ZO-1.

### 2.8 Measurement of gut permeability using FITC–dextran assay

Mice were fasted for 4 h before receiving an oral gavage of FD4 solution (60 mg per 100 g body weight, prepared in PBS). Four hours post-gavage, blood was collected via retro-orbital bleeding and centrifuged at 3000×*g* for 15 min to obtain plasma. Fluorescence intensity was measured using a microplate reader at an excitation wavelength of 485 nm and emission at 528 nm.

### 2.9 Serum zonulin quantification by ELISA

Whole blood was collected from mice, allowed to clot at room temperature, and centrifuged to obtain serum. Zonulin levels in the resulting serum samples were measured using a Mouse Zonulin ELISA Kit (UpingBio, Wuhan, China) following the manufacturer's instructions.

### 2.10 Immunofluorescence detection of intestinal tight junctions

Ileum and colon tissues were fixed in 4 % paraformaldehyde, embedded in paraffin, and sliced into 5 μm sections. Following standard dewaxing and antigen retrieval procedures, tissue sections were blocked and incubated overnight at 4 °C with primary antibodies targeting Claudin-1, Occludin, and ZO-1 (all from Servicebio, Wuhan, China; dilution 1:100). The next day, sections were treated with Alexa Fluor-labeled secondary antibodies (Invitrogen, CA, USA) at room temperature for 1 h. Nuclei were counterstained with DAPI, and fluorescence images were captured using a Zeiss LSM 880 confocal microscope (Germany). Fluorescence intensity was quantitatively analyzed using ImageJ software. All quantitative analyses of fluorescence intensity were performed in a blinded manner with respect to the experimental groups.

### 2.11 Histological assessment of intestinal inflammation

Ileal and colonic tissues were harvested and fixed in 4 % paraformaldehyde. Samples were embedded in paraffin, cut into 5 μm sections, and stained with hematoxylin and eosin (H&E) or periodic acid–Schiff (PAS) reagents (Servicebio, Wuhan, China) according to standard protocols. Stained slides were examined under a light microscope, and digital images were acquired using the Pannoramic MIDI slide scanning system. Histopathological scoring for inflammation and mucosal damage was conducted by two independent observers who were blinded to the group allocation of the samples.

### 2.12 Evaluation of combined CG@GelMA and TAA treatment

Following successful induction of rheumatoid arthritis, mice that developed arthritis (n = 20) were randomly assigned on day 25 into four treatment groups: (i) Control group, receiving oral ddH_2_O and intra-articular PBS; (ii) CG@GelMA group, receiving daily oral administration of CG@GelMA (300 μL containing 1.4 × 10^7^ CV cells/mL and 2 mg/mL GPS); (iii) TAA group, receiving oral ddH_2_O and intra-articular injections of triamcinolone acetonide (5 μL of 2 mg/mL) on days 30, 36, 42, and 48; and (iv) CG@GelMA + TAA group, receiving both treatments according to the above schedules. Arthritis index scores and hind paw photographs were recorded every five days from day 21 to day 49, while locomotor performance—assessed via stride and step length—was evaluated on day 49. Intestinal permeability was measured on day 48 by quantifying serum FITC-dextran levels after oral gavage. On day 50, mice were euthanized for endpoint analyses. Joints were collected for H&E, TRAP, and Safranin O/Fast Green staining to assess inflammation, bone erosion, and cartilage integrity. Intestinal tissues were harvested for length measurement, histological analysis, immunofluorescence staining of Claudin-1, Occludin, and ZO-1, and tight junction gene expression by qPCR. Systemic immune responses were evaluated by measuring serum levels of IL-10 and IL-17, as well as by analyzing splenic Treg and Th17 cells via flow cytometry. In addition, fecal samples were collected for 16S rDNA sequencing to profile gut microbiota composition and explore microbial shifts associated with treatment.

### 2.13 Histological evaluation of bone and joint damage

Tibiotarsal (ankle) joints were fixed in 4 % paraformaldehyde for 24 h and then decalcified using a commercial decalcifying agent (Servicebio, Wuhan, China) following the manufacturer's protocol. Tissues were processed into paraffin blocks, and 2 μm sections were prepared. These were stained with H&E, tartrate-resistant acid phosphatase (TRAP), and Safranin O/Fast Green (SO/FG) using standard staining kits from Servicebio.

### 2.14 Flow cytometric analysis of splenic immune cells

Single-cell suspensions were prepared from mouse spleens for flow cytometric analysis. Spleens were gently disrupted using frosted glass slides, and the resulting cell suspensions were filtered through 70 μm nylon meshes. Cells were then centrifuged at 400×*g* for 5 min to obtain mononuclear cells. Regulatory T cells (Tregs) and Th17 cells were subsequently stained using commercial kits specific for each cell type (Mouse Regulatory T Cell Staining Kit and Mouse Th17 Staining Kit; MultiSciences (Lianke), Hangzhou, China), in accordance with the manufacturer's instructions.

### 2.15 Quantitative real-time PCR

Total RNA was extracted from dissected colon tissues using TRIzol reagent (Invitrogen, USA) according to the manufacturer's protocol. Tissue samples were thoroughly homogenized in TRIzol to ensure complete lysis. Reverse transcription was performed using ABScript III RT Master Mix with gDNA Remover (ABclonal, RK20429) in a total volume of 20 μL. Quantitative PCR was carried out using 2 × Universal SYBR Green Fast qPCR Mix (ABclonal, Wuhan, China) on a QuantStudio™ Real-Time PCR System (Applied Biosystems, USA). The relative mRNA expression levels of Claudin-1, Occludin, and ZO-1 were quantified, with GAPDH serving as the internal reference gene. The 2^∧^−ΔΔCT method was employed to calculate fold changes in gene expression relative to the control group.

### 2.16 Microbial community analysis

Fecal microbial DNA was extracted from mouse stool samples using the Qubit dsDNA HS Assay Kit, followed by 16S rDNA sequencing. Sequencing data were used to assess microbial composition and diversity. Sample coverage and sequencing depth were evaluated using the Goods_coverage index to ensure data reliability. Beta diversity was examined through principal coordinates analysis (PCoA) based on Bray–Curtis distance matrices, allowing for visualization of intergroup differences in microbial community structure. Taxonomic composition at both the phylum and genus levels was profiled, and the top 20 genera in relative abundance were presented. Microbial network analysis was conducted to explore co-occurrence relationships among taxa. Differential abundance across groups was identified using metagenomeSeq and LEfSe (Linear Discriminant Analysis Effect Size), enabling the identification of specific microbial signatures associated with disease status or therapeutic interventions. For the visualization of key microbial taxa in heatmaps and the presentation of relative abundance comparisons, we followed established methods from recent literature to enhance clarity and interpretability [64].

### 2.17 Fecal microbiota transplantation (FMT) and microbiota dependence

To determine whether the therapeutic benefits of CG@GelMA are mediated via gut microbiota, fecal microbiota transplantation (FMT) was performed using feces collected from CG@GelMA – treated donors. CIA mice with established arthritis were assigned to two groups: the FMT group received freshly prepared fecal suspensions (200 mg/mL in PBS; 300 μL per dose, every other day from day 25–49), The ABX + FMT group received freshly prepared fecal suspensions in parallel with broad-spectrum antibiotics (streptomycin 2.5 mg/mL, ampicillin 0.3 mg/mL, colistin 0.3 mg/mL) administered via sterile drinking water from day 25–49, with water refreshed three times per week to prevent stable microbiota colonization, and the FMT (Inactivated) group received suspensions that had been sterilized by autoclaving (121 °C, 20 min) to abolish microbial viability. Arthritis scores, body weight, and hind paw photographs were recorded every 4 days throughout the treatment period. On day 48, intestinal permeability was assessed by serum FITC–dextran following oral gavage. On day 49, locomotor performance was evaluated. All mice were euthanized on day 50 for endpoint analyses, including joint histology, intestinal morphology, and splenic immune profiling, and joint damage was assessed by Micro-CT and histology, while intestinal length and pathology were recorded. Splenic T cell subsets were analyzed by flow cytometry to evaluate systemic immunomodulatory effects of microbiota transfer.

### 2.18 Oral biosafety evaluation

Female Balb/c mice (6 weeks old) were randomly allocated into five groups: Control, CV, GPS, Blank@GelMA, and CG@GelMA. For 30 consecutive days, each group received a daily intragastric administration of 300 μL of their respective treatment: PBS, CV (1.4 × 10^7^ cells/mL), GPS (2 mg/mL), Blank@GelMA (2.5 mg/mL), or CG@GelMA (CV = 1.4 × 10^7^ cells/mL and GPS = 2 mg/mL). Upon completion of the treatment period, the mice were euthanized. Blood samples were then collected for hematological and biochemical analyses, while major organs (heart, liver, spleen, lungs, kidneys, stomach, and intestines) were harvested for histological examination via H&E staining.

### 2.19 Statistical analysis

Data are expressed as mean ± standard deviation (SD). The normality of data distribution was evaluated using the Shapiro–Wilk test. For datasets that followed a normal distribution, comparisons between two groups were performed with the Student's t-test. Comparisons involving a single factor were analyzed using one-way ANOVA, whereas data involving two independent variables were analyzed by two-way ANOVA, followed by Dunnett's post-hoc test for comparisons with the control group. In cases where data were not normally distributed, non-parametric tests were employed: the Mann–Whitney *U* test for two-group comparisons and the Kruskal–Wallis test for multiple groups. All statistical analyses were conducted using GraphPad Prism (version 8.0), with Microsoft Excel 2016 used for complementary data organization. A *p*-value ≤0.05 was considered statistically significant, denoted as ∗*p* ≤ 0.05, ∗∗*p* ≤ 0.01, and ∗∗∗*p* ≤ 0.001.

## 3 Results

### 3.1 Fabrication and characterization of thermoresponsive microspheres

**Fig. 1:**
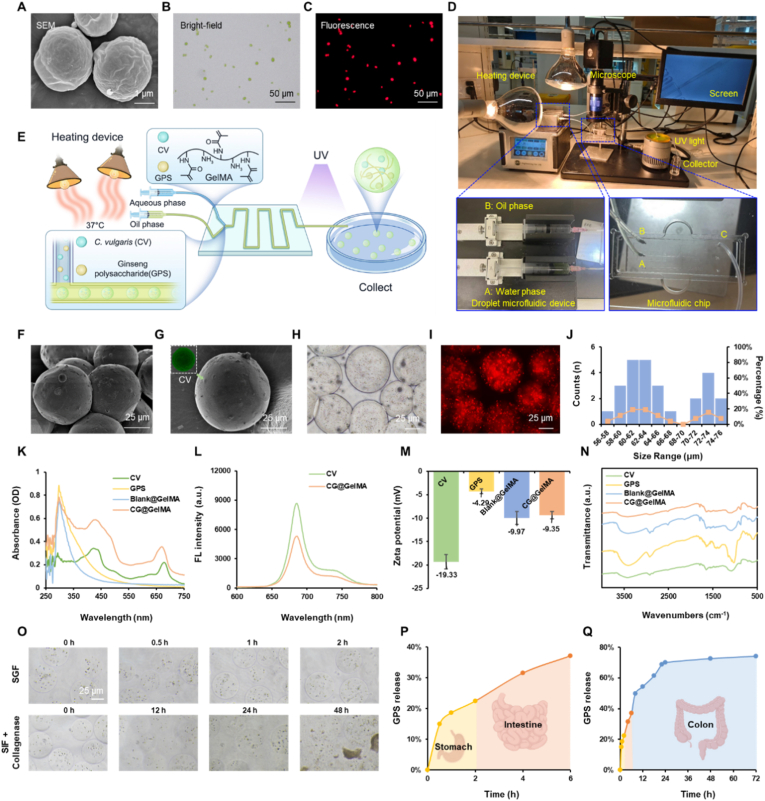
Fabrication and characterization of CG@GelMA. (A) SEM image of *Chlorella vulgaris* (CV). Scale bar: 1 μm. (B–C) Bright-field (B) and Fluorescence (C) microscopy images of CV. Scale bars: 50 μm. (D) Photograph of the microfluidic device, including droplet generator, microfluidic chip, microscope, heating unit, screen, UV light source, and collector. The aqueous phase was injected into channel A, the oil phase into channel B, and microdroplets were collected from channel C. (E) Schematic illustration of the microfluidic process for generating monodisperse water-in-oil microspheres. (F–G) SEM images of CG@GelMA. Scale bars: 25 μm. (H–I) Bright-field (H) and Fluorescence (I) microscopy images of CG@GelMA. Scale bars: 25 μm. (J) Size distribution of CG@GelMA. (K) UV–vis absorption spectra of CV, GPS, Blank@GelMA, and CG@GelMA. (L) Fluorescence emission spectra of CV and CG@GelMA under excitation at 552 nm. (M) Zeta potentials of CV, GPS, Blank@GelMA, and CG@GelMA. (N) FTIR spectra of CV, GPS, Blank@GelMA, and CG@GelMA. (O) Morphological changes of CG@GelMA in simulated gastric fluid (SGF, pH 1.2) and simulated intestinal fluid (SIF, pH 6.8, with collagenase). (P) Cumulative release profiles of GPS from CG@GelMA in SGF and SIF. (Q) Cumulative release profile of GPS in simulated colonic fluid. Data are presented as means ± SD.

Scanning electron microscopy (SEM) revealed that CV exhibited a well-defined spherical morphology with a mean diameter of approximately 2 μm (Fig. 1A). Bright-field microscopy showed the characteristic green coloration of CV, while fluorescence imaging confirmed its strong red autofluorescence (Fig. 1B–C), validating the structural integrity and excellent intrinsic fluorescence properties of CV. To achieve targeted and thermoresponsive oral delivery, a microfluidic platform was utilized to fabricate GelMA hydrogel microspheres co-loaded with CV and GPS (Fig. 1D). This system precisely controlled the two-phase flow by co-injecting an aqueous phase—composed of methacrylated gelatin (GelMA), the photoinitiator lithium phenyl-2,4,6-trimethylbenzoylphosphinate (LAP), CV, and GPS—with an oil phase (liquid paraffin containing Span 80) into a microfluidic chip. Interfacial tension and shear forces segmented the aqueous phase into monodisperse droplets (Fig. S1). Due to the thermosensitive nature of GelMA, the entire process was maintained at ∼37 °C to ensure low viscosity and stable flowability of the aqueous mixture, thus promoting consistent droplet formation. Subsequently, these droplets were rapidly crosslinked under UV irradiation (365 nm, 80 mW/cm^2^), yielding structurally stable CV/GPS-loaded microspheres (CG@GelMA) (Fig. 1E).

SEM imaging of the resulting CG@GelMA revealed uniform spherical morphology with CV visibly embedded on the microsphere surface, supporting successful encapsulation (Fig. 1F and G). Bright-field (Fig. 1H and Fig. S2A–B) and fluorescence microscopy (Fig. 1I and Fig. S1C–D) showed the uniform presence of CV and its characteristic red autofluorescence within the GelMA microspheres, demonstrating successful loading and enabling optical visualization. Size distribution analysis indicated that the microspheres ranged from 56 to 76 μm in diameter, with the majority (38.5 %) falling within the 60–64 μm range, reflecting good uniformity and suitability for oral delivery applications (Fig. 1J).

UV–vis and fluorescence spectroscopy revealed characteristic absorption and emission peaks corresponding to both CV and GPS, which were retained in CG@GelMA, indicating preservation of their native optical properties after encapsulation (Fig. 1K–L). Zeta potential analysis showed that CG@GelMA (−9.35 mV) had a less negative charge than free CV (−19.33 mV) and GPS (−4.29 mV), and was close to Blank@GelMA (−9.97 mV), suggesting partial charge neutralization upon encapsulation within the hydrogel (Fig. 1M). FTIR analysis confirmed the successful incorporation of CV and GPS into the GelMA microspheres without altering the hydrogel's structural integrity (Fig. 1N). To demonstrate the structural recovery capacity of the microspheres, lyophilized CG@GelMA were rehydrated in PBS. Remarkably, the collapsed microspheres gradually restored their spherical architecture within 12h (Fig. S3). Next, to assess their performance in physiologically relevant environments, we evaluated the swelling, degradation, and release behavior of the microspheres in simulated gastrointestinal fluids at 37 °C. The microspheres maintained structural integrity with minimal swelling in simulated gastric fluid (SGF, pH 1.2) during the first 2 h. Upon transfer to simulated intestinal fluid (SIF, pH 6.8, supplemented with collagenase), they gradually swelled and degraded from 12 h onward, with nearly complete degradation observed at 48 h, highlighting their responsiveness to enzymatic conditions in the intestinal environment (Fig. 1O).

To simulate the digestive environment, the release behavior of CG@GelMA was tested *in vitro* using SGF, SIF, and simulated colonic fluid (SCF). Drug concentrations in the release media were quantified by UV–visible spectroscopy, referencing a GPS standard curve established for each fluid type (Fig. S4). GPS release from CG@GelMA microspheres was limited in SGF, with only 22.5 % released within the first 2 h, indicating good stability under acidic gastric conditions. Upon transition to SIF, the release increased to 31.9 % at 6 h, followed by a more pronounced release in simulated colonic fluid, with cumulative release reaching 71.8 % at 72 h (Fig. 1P–Q).

In summary, CG@GelMA microspheres display uniform spherical morphology and significant encapsulation as evidenced by microscopy, along with notable structural recovery ability after dehydration. The preservation of CV's intrinsic red autofluorescence supports potential for *in vivo* imaging. Additionally, the microspheres show strong acid resistance and controlled, sustained drug release, with limited release in gastric conditions and gradually increased release in intestinal and colonic environments. These features indicate their promise as an oral delivery platform with enhanced intestinal bioavailability and immunomodulatory potential.

### 3.2 CG@GelMA protects intestinal epithelial barrier integrity against FSN-induced impairment in vitro

**Fig. 2:**
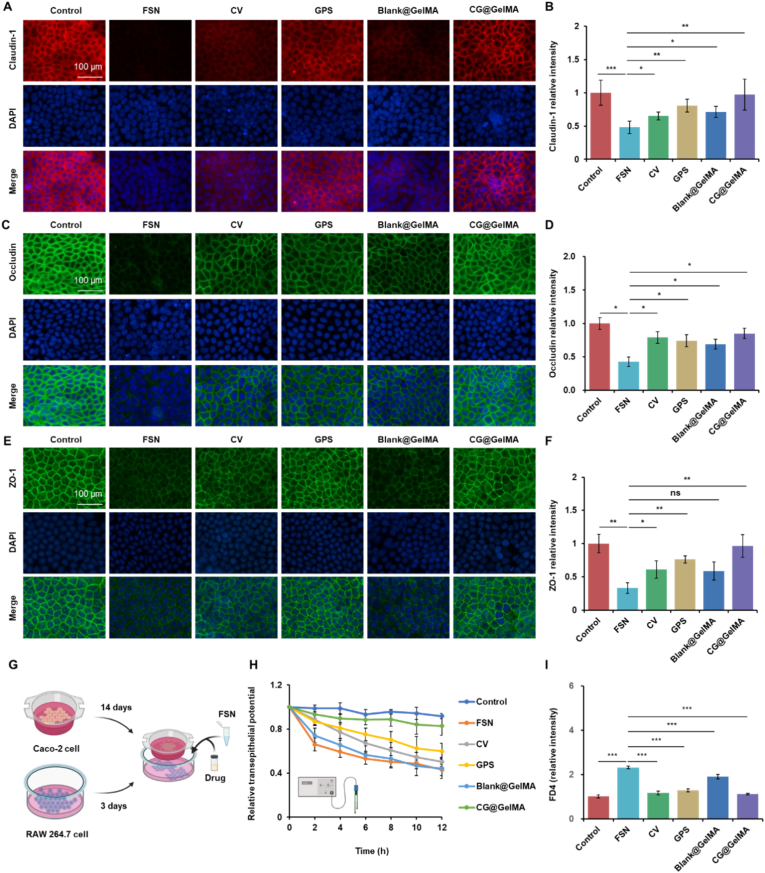
Evaluation of the protective effects of CG@GelMA on FSN-induced intestinal epithelial barrier disruption *in vitro*. (A) Immunofluorescence staining of Claudin-1 in Caco-2 monolayers under different treatments. Scale bar: 100 μm. (B) Relative fluorescence intensity of Claudin-1. (C) Immunofluorescence staining of Occludin in Caco-2 monolayers. Scale bar: 100 μm. (D) Relative fluorescence intensity of Occludin. (E) Immunofluorescence staining of ZO-1 in Caco-2 monolayers. Scale bar: 100 μm. (F) Relative fluorescence intensity of ZO-1. (G) Schematic diagram of the Caco-2/RAW 264.7 Transwell co-culture system. (H) Relative TEER of Caco-2 cell monolayers after different treatments. (I) Relative fluorescence intensity of FD4 across Caco-2 monolayers under different treatments. Data are presented as means ± SD. Statistical significance: ∗*P* < 0.05, ∗∗*P* < 0.01, ∗∗∗*P* < 0.001.

To evaluate the protective effects of CG@GelMA on the intestinal epithelial barrier under conditions mimicking rheumatoid arthritis (RA)-associated gut dysbiosis, we utilized an *in vitro* model challenged with fecal supernatant (FSN) derived from collagen-induced arthritis mice which served as a disease-relevant positive control. We first evaluated the effects of FSN on tight junction integrity in Caco-2 monolayers and the potential protective roles of CV, GPS, Blank@GelMA, and CG@GelMA. Immunofluorescence staining revealed that FSN exposure markedly disrupted the localization and fluorescence intensity of Claudin-1, Occludin, and ZO-1 at cell junctions (Fig. 2A–C, E), which was further confirmed by quantitative analysis (Fig. 2B–D, F).

Western blot analysis further confirmed that CG@GelMA most effectively restored the expression of Occludin and ZO-1 at the protein level, outperforming its individual components and Blank@GelMA (Fig. S5). In addition to structural integrity, maintaining the functional integrity of the intestinal barrier is critical for preserving homeostasis. Therefore, we assessed barrier integrity using an epithelial-macrophage co-culture system (Fig. 2G). In this model, Caco-2 cells were differentiated into polarized monolayers in the upper chamber of Transwell inserts, while RAW264.7 macrophages were cultured in the basal compartment. Macrophages were pretreated with CV, GPS, Blank@GelMA, or CG@GelMA for 1 h before stimulation with FSN, simulating the exposure of gut immune cells to RA-associated luminal inflammatory mediators. We then monitored the functional consequences of FSN stimulation on the epithelial barrier integrity. Stimulation of the basal macrophages with FSN induced a rapid and significant decline in transepithelial electrical resistance (TEER) over 12 h, indicating compromised barrier function. Pretreatment of macrophages with CV, GPS, Blank@GelMA, or CG@GelMA attenuated this FSN-induced TEER reduction. CV and GPS pretreatment provided significant protection compared to FSN treatment. Blank@GelMA pretreatment showed a weaker protective effect. Importantly, pretreatment with CG@GelMA demonstrated the strongest protective effect, maintaining TEER values significantly higher than those in the FSN group and comparable to the untreated control (Fig. 2H). Additionally, to unequivocally determine whether the enhanced barrier protection observed with the CV and GPS combination was additive or truly synergistic, we calculated the theoretical additive TEER value. The measured TEER value for the CV + GPS group significantly exceeded this theoretical additive value (Fig. S6), confirming a synergistic interaction between CV and GPS in restoring barrier function.

To directly assess macromolecule permeability across the epithelial monolayer, we measured the fluorescence intensity of fluorescein isothiocyanate-dextran 4 kDa (FD4) after a 2-h incubation. Consistent with the TEER results, FSN stimulation significantly increased the fluorescence intensity of FD4 in the basolateral chamber, indicating elevated paracellular permeability. Pretreatment with CV, GPS, Blank@GelMA, or CG@GelMA significantly attenuated this FSN-induced increase. CV and GPS treatment both reduced FD4 permeability, while Blank@GelMA had a modest effect. Notably, CG@GelMA exhibited the most pronounced protective effect, reducing permeability to levels comparable to the control group (Fig. 2I). Beyond barrier protection, the cytoprotective and antioxidant effects of CG@GelMA were evaluated in IEC-6 cells subjected to inflammatory stress. Lipopolysaccharide (LPS) treatment induced oxidative damage, evidenced by a marked increase in intracellular reactive oxygen species (ROS) detected via DCFH-DA staining. Treatment with CV, GPS, Blank@GelMA, or CG@GelMA significantly reduced ROS levels, with CG@GelMA showing the strongest inhibitory effect (Fig. S7). Additionally, Calcein-AM/PI staining demonstrated that CG@GelMA substantially decreased the proportion of PI-positive dead cells following LPS exposure, indicating robust protection against inflammation-induced cytotoxicity (Fig. S8). Consistent with these findings, CCK-8 assays revealed that all treatments improved cell viability in a dose-dependent manner (Fig. S9). To elucidate the underlying mechanism of barrier protection, we analyzed macrophage polarization. Flow cytometry revealed that CG@GelMA most effectively suppressed the LPS-induced shift towards a pro-inflammatory M1 phenotype (Fig. S10). This immunomodulatory effect contributes to its superior efficacy in preserving epithelial barrier integrity.

Collectively, these findings demonstrate that CG@GelMA effectively protects the intestinal epithelial barrier against FSN-induced damage. This protective effect was evident both structurally, through the preservation of key tight junction proteins (Claudin-1, Occludin, ZO-1), and functionally, by maintaining high TEER and limiting macromolecular permeability, as indicated by reduced FD4 fluorescence. Notably, CG@GelMA exhibited superior efficacy compared to its individual components (CV, GPS) and Blank@GelMA, suggesting a synergistic advantage in maintaining gut barrier integrity under inflammatory conditions. Together, these results indicate that CG@GelMA exerts dual protective functions by enhancing epithelial barrier integrity and alleviating oxidative stress in intestinal epithelial cells, underscoring its therapeutic potential for mitigating RA-associated gut barrier dysfunction. Future validation in more physiologically relevant systems, such as intestinal organoids, is planned to further elucidate the mechanisms within a complex tissue context.

### 3.3 Anti-inflammatory and antioxidant effects of CG@GelMA in vitro

To evaluate the anti-inflammatory and antioxidant capacities of CG@GelMA under inflammatory conditions, we first analyzed its effect on the expression of key pro-inflammatory cytokines in an LPS-stimulated inflammation model using IEC-6 cells. As shown in Fig. S11, compared to the LPS-induced model group, CV treatment significantly reduced the mRNA expression levels of the pro-inflammatory cytokines IL-6, IL-1β, and TNF-α. In contrast, GPS treatment showed no significant effect on IL-6 and IL-1β levels, and the Balnk@GelMA did not significantly inhibit any of the tested inflammatory cytokines. However, when CV and GPS were used in combination (in both the CV + GPS group and the CG@GelMA group), the levels of inflammatory cytokines were further significantly reduced compared to the CV treatment alone, clearly indicating a synergistic anti-inflammatory effect between the two components.

We further quantified the direct antioxidant activity of CG@GelMA by assessing its capacity to scavenge three key reactive oxygen species. The scavenging effects on hydroxyl radicals (·OH), hydrogen peroxide (H_2_O_2_), and superoxide anions (O_2_·^-^) were determined using the salicylic acid, TMB, and NBT assays, respectively (Figs. S12–S14). All treatments containing CV (*i.e.*, CV, CV + GPS, and CG@GelMA) exhibited a concentration-dependent increase in scavenging activity against all three ROS. In contrast, GPS and Blank@GelMA treatments showed only minimal, non-concentration-dependent effects. Critically, at every concentration tested, the CV + GPS combination and CG@GelMA demonstrated significantly greater scavenging efficiency than CV alone. These results clearly indicate that while GPS has limited intrinsic antioxidant capacity, it acts synergistically with CV to potentiate its reactive oxygen species scavenging efficacy.

Collectively, these *in vitro* results demonstrate that CG@GelMA not only effectively suppresses the expression of pro-inflammatory cytokines through a synergistic interaction but also synergistically enhances the scavenging capacity for multiple ROS, together constituting its dual protective mechanism against inflammatory and oxidative damage.

### 3.4 In vivo biodistribution, biodegradation, and excretion of CG@GelMA

**Fig. 3:**
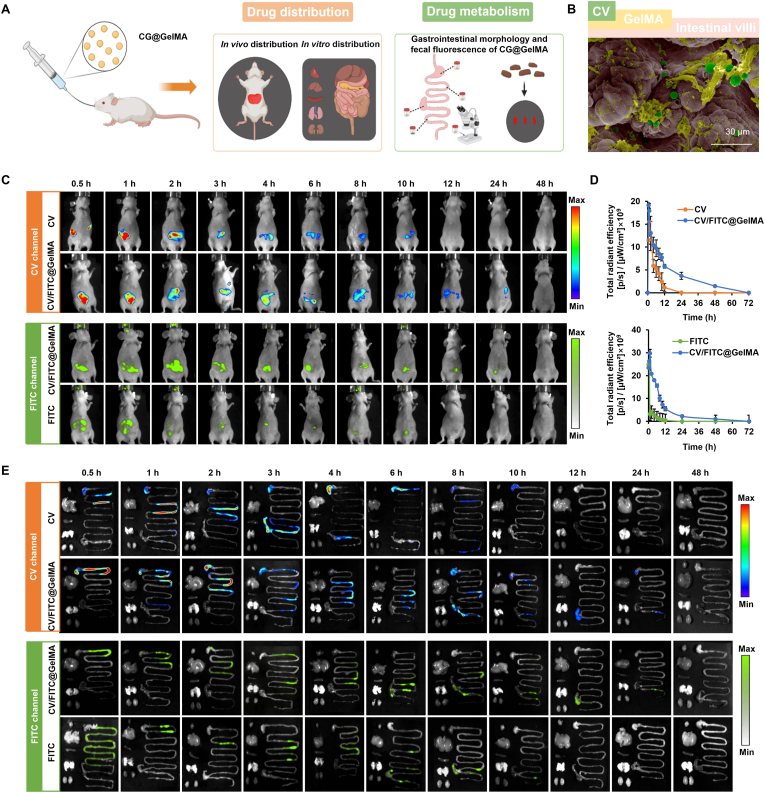
*In vivo* biodistribution and gastrointestinal transit of CG@GelMA. (A) Schematic of the experimental procedure: oral gavage of CG@GelMA, followed by *in vivo* fluorescence imaging, *ex vivo* organ distribution, microscopic analysis of gastrointestinal contents from different intestinal segments (stomach, ileum, cecum, and colon), and fecal fluorescence monitoring. (B) SEM image of CG@GelMA adhered to intestinal villi. Pseudocolor rendering distinguishes CV (green), GelMA (yellow), and intestinal villi (pink). Scale bar: 30 μm. (C) Time-dependent *in vivo* fluorescence imaging of mice following oral administration of CV, FITC, or CV/GPS@GelMA, captured under the CV (chlorophyll) and FITC channels. (D) Quantification of abdominal fluorescence intensity among different treatment groups. (E) *Ex vivo* fluorescence imaging of major organs (heart, liver, spleen, lung, kidney, stomach, small intestine, and large intestine) at different time points. Data are presented as means ± SD.

To investigate the *in vivo* distribution and degradation of CG@GelMA, we conducted real-time fluorescence imaging using the IVIS Spectrum system. Chlorophyll in CV exhibits intrinsic near-infrared autofluorescence (∼680 nm), which facilitated direct visualization of CV. Additionally, FITC was employed as a green fluorescent surrogate for GPS due to its spectral distinction from chlorophyll, enabling visualization of its distribution. As illustrated in Fig. 3A, *in vivo* and *ex vivo* imaging were used to assess gastrointestinal distribution. SEM imaging showed that CV and GelMA adhered to the surface of intestinal villi after oral administration of CG@GelMA (Fig. 3B). Whole-body imaging revealed that CG@GelMA exhibited prolonged retention in the gastrointestinal tract, with persistent fluorescence signals observed in the abdominal region for over 24 h. In contrast, signals from CV or FITC alone diminished significantly within this period (Fig. 3C). At 24 h post-administration, the fluorescence intensity in the CG@GelMA group was significantly higher than that in the CV and FITC groups, with red and green signals being 3.2-foldand 1.67-fold higher, respectively (Fig. 3D).

Consistent with *in vivo* imaging results, CG@GelMA exhibited stronger and more sustained fluorescence signals in the gastrointestinal tract compared to CV or FITC. Fluorescence remained detectable within the gastrointestinal tract up to 24 h post-administration. Notably, fluorescence was confined to the gastrointestinal tract, with no detectable signal in other major organs, indicating minimal systemic distribution (Fig. 3E). SEM analysis of luminal contents confirmed that CG@GelMA maintained structural integrity in the stomach, progressively degraded in the small intestine, and was fully disintegrated in the colon, indicating its ability to resist gastric conditions and achieve sustained intestinal degradation (Fig. S15). Additionally, time-dependent fluorescence signals in fecal samples confirmed the effective excretion of CG@GelMA after administration (Fig. S16).

### 3.5 Intestinal barrier dysfunction in arthritis susceptibility and progression

**Fig. 4:**
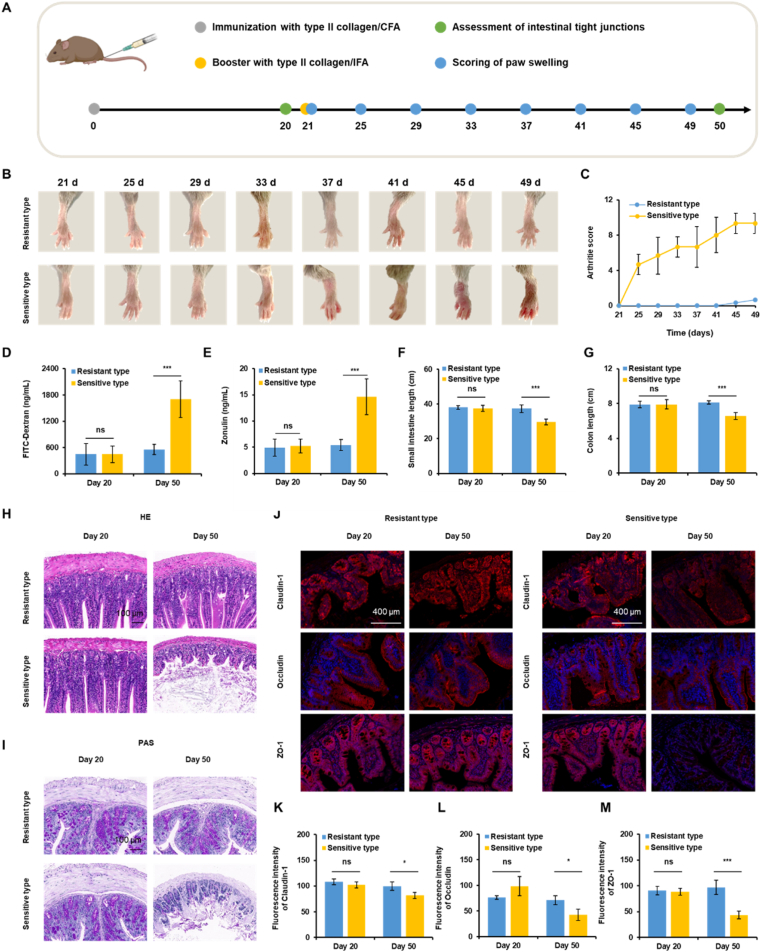
Association between intestinal barrier dysfunction and arthritis susceptibility. (A) Schematic diagram depicting the overall experimental design, including arthritis induction, subgroup classification, and intestinal barrier assessments. (B) Representative paw images of sensitive and resistant CIA mice from day 21 to day 49. (C) Arthritis scores monitored over time from day 21 to day 49. (D) Serum FITC-dextran levels on day 20 and day 50. (E) Serum zonulin levels on day 20 and day 50. (F) Small intestinal length measured on days 20 and 50. (G) Colonic length measured on days 20 and 50. (H–I) H&E (H) and PAS (I) staining of ileum tissues on day 20 and day 50. Scale bars: 100 μm. (J) Immunofluorescence staining for Claudin-1, Occludin, and ZO-1 in ileum tissues on day 20 and day 50. Scale bars: 400 μm. (K–M) Quantification of Claudin-1 (K), Occludin (L), and ZO-1 (M) fluorescence intensity of ileum tissues on day 20 and day 50. Data are presented as means ± SD. Statistical significance: ∗*P* < 0.05, ∗∗*P* < 0.01, ∗∗∗*P* < 0.001.

To explore the relationship between arthritis development and intestinal barrier integrity, mice were induced with CIA. Starting from day 21, paw swelling was photographed and assessed every four days to monitor disease progression. On day 25, mice were classified into sensitive and resistant groups based on joint inflammation severity. Intestinal tight junction status was evaluated at both day 20 and day 50 to assess changes in gut barrier function over the course of disease (Fig. 4A). Beginning on day 25, sensitive-type mice exhibited progressive paw swelling, while resistant-type mice showed no visible signs of redness or swelling throughout the observation period (Fig. 4B). Consistently, arthritis scores in the sensitive-type group increased steadily through day 49, whereas the resistant-type group maintained low and non-progressive scores, suggesting limited clinical symptoms and minimal disease progression (Fig. 4C). Histological analysis revealed no pathological alterations in the joints of resistant-type mice at either day 25 or day 50. In contrast, sensitive-type mice developed severe destructive arthritis pathology by day 50 (Fig. S17).

To assess the systemic impact of arthritis, body weight and spleen size were monitored. By day 49, mice in the sensitive-type group exhibited lower body weight compared to the resistant type, reflecting increased systemic burden associated with disease progression (Fig. S18). In line with this, spleen weights were comparable between groups at day 20; however, by day 50, sensitive type mice exhibited significant splenomegaly, indicative of heightened systemic inflammatory response (Fig. S19). To determine whether intestinal barrier integrity is associated with arthritis susceptibility and disease progression, we evaluated gut permeability on day 20 and day 50. On day 20, no significant differences in serum FITC-dextran or zonulin levels were detected between mice that later developed arthritis (sensitive type) and those that did not (resistant type) (Fig. 4D and E). By day 50, however, both FITC-dextran and zonulin levels were markedly elevated in the sensitive type, indicating progressive intestinal barrier disruption concurrent with disease severity. Morphological assessment further revealed significant shortening of both the small intestine and colon in sensitive type mice at day 50, whereas no differences were evident at day 20 (Fig. 4F and G). Histological staining (H&E and PAS) confirmed normal intestinal architecture in both groups at day 20. In contrast, by day 50, sensitive type mice exhibited evident mucosal injury, including villus blunting and crypt disorganization, along with reduced goblet cell numbers. Corroborating these histopathological findings, the clinical arthritis scores in this group showed a parallel progressive worsening (Fig. 4H, I, Fig. S20–S21).

To directly assess the integrity of intestinal tight junctions, immunofluorescence staining of ileum and colonic tissues was performed. At day 20, both groups displayed strong and continuous expression of Claudin-1, Occludin, and ZO-1. However, by day 50, expression of all three markers was markedly diminished and disrupted in the sensitive type group (Fig. 4J and Fig. S22). Quantitative analysis confirmed significantly reduced fluorescence intensity for each tight junction protein compared to the resistant type on day 50 (Fig. 4K–M and Fig. S23A–C). These findings demonstrate that the development and progression of arthritis are accompanied by marked intestinal barrier dysfunction, as evidenced by increased permeability, disrupted tight junction architecture, intestinal shortening, and mucosal damage. The observed differences in intestinal barrier function between resistance type and sensitive type suggest a close association between gut barrier integrity and arthritis severity. These results support the existence of a temporal correlation between intestinal barrier disruption and joint inflammation, indicating that gut barrier status may be relevant to the clinical course of RA.

### 3.6 Combined treatment ameliorates arthritis and improves joint function

**Fig. 5:**
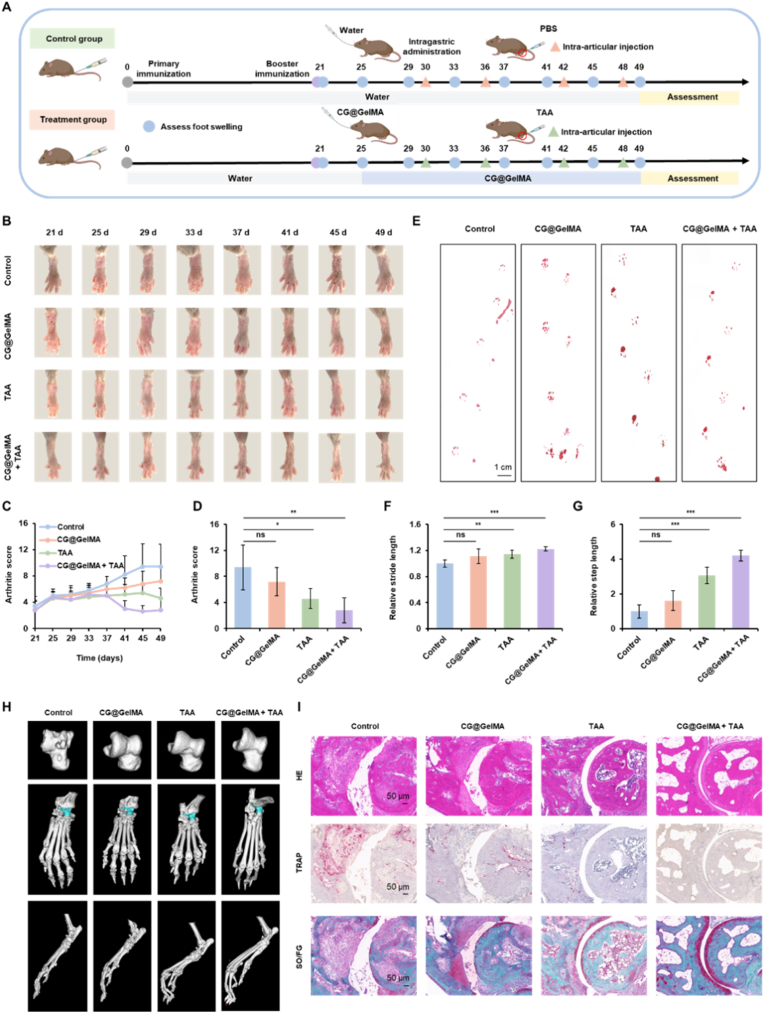
Therapeutic efficacy of combined oral CG@GelMA and intra-articular TAA treatment in CIA mice. (A) Schematic illustration of the experimental design showing timelines for CIA induction, oral CG@GelMA administration (starting day 25), and intra-articular TAA injection (starting day 30), along with joint and intestinal assessments. (B) Representative images of hind paws from different treatment groups captured between day 21 and day 49. (C–D) Clinical arthritis scores of different groups monitored over time (C) and quantified at day 49 (D). (E) Representative gait diagrams recorded by footprint analysis. Scale bars: 1 cm. (F–G) Quantification of relative stride length (F) and step length (G) in different treatment groups. (H) Representative 3D micro-CT reconstructions of hind limb joints from each group. (I) Histological assessment of joints by H&E, TRAP, and Safranin O/Fast Green staining. Scale bars: 50 μm. Data are presented as means ± SD. Statistical significance: ∗*P* < 0.05, ∗∗*P* < 0.01, ∗∗∗*P* < 0.001.

Given the interplay between intestinal dysfunction and joint inflammation, we aimed to investigate whether a combined treatment strategy—oral administration to restore gut homeostasis together with intra-articular therapy—could provide superior therapeutic efficacy in RA. We employed a CIA mouse model, initiating oral administration on day 25 after primary immunization, followed by intra-articular injection starting on day 30 (Fig. 5A). Hind paw images collected between day 21 and day 49 revealed progressive joint swelling in the control group. Mice treated with CG@GelMA group exhibited a modest reduction in swelling, while the TAA group provided greater relief. Notably, the CG@GelMA + TAA group demonstrated the most pronounced amelioration of joint appearance (Fig. 5B). These visual improvements were supported by clinical arthritis scores, which indicated ongoing disease progression in the Control group, partial alleviation in the CG@GelMA group and TAA group, and the most effective symptom control in the CG@GelMA + TAA group (Fig. 5C and D).

Locomotor performance was assessed to evaluate the therapeutic effects of the combined treatment on arthritis. The Control group exhibited marked gait impairment, as evidenced by reduced relative stride and step lengths. Treatment with the CG@GelMA group exerted only mild effects on gait restoration. In contrast, the TAA group resulted in a clear improvement in gait performance. Notably, the CG@GelMA + TAA group exhibited the most pronounced enhancement in both relative stride length and step length, indicating a more effective recovery of joint mobility and overall locomotor function (Fig. 5E–G). Micro-CT analysis of the hind limbs further substantiated the therapeutic efficacy. Severe bone erosion and joint deformities were observed in the Control group. Both the CG@GelMA group and the TAA group exhibited incomplete bone erosion, while the CG@GelMA + TAA group demonstrated the most well-preserved joint architecture, indicating a synergistic protective effect. Consistently, quantitative analysis of bone parameters (BMD, BV/TV, Tb.N, and Tb.Th) showed marked increases in the CG@GelMA + TAA group compared to the Control group (Fig. 5H and Fig. S24).

H&E staining revealed substantial inflammatory infiltration in the Control group. These features were attenuated in the CG@GelMA group and TAA group, and were most prominently improved in the CG@GelMA + TAA group. TRAP staining indicated a reduction in osteoclast number following all treatments, with the lowest level observed in the CG@GelMA + TAA group. Safranin O/Fast Green staining showed that cartilage degradation was markedly ameliorated by CG@GelMA + TAA group treatment, compared to partial protection by CG@GelMA group and TAA group (Fig. 5I). These results collectively demonstrate that combined oral and intra-articular treatment with CG@GelMA and TAA effectively mitigates joint inflammation, preserves cartilage and bone structures, and improves motor function in arthritic mice. To further verify the specific roles of each component, a supplementary CIA mouse experiment confirmed that only the co-encapsulated CG@GelMA + TAA formulation exerted significant therapeutic effects on joint swelling and arthritis scores, whereas individual components or their physical mixture showed no superiority over the control group (Figs. S25–S26)

### 3.7 Restores intestinal barrier integrity and modulates immune responses

**Fig. 6:**
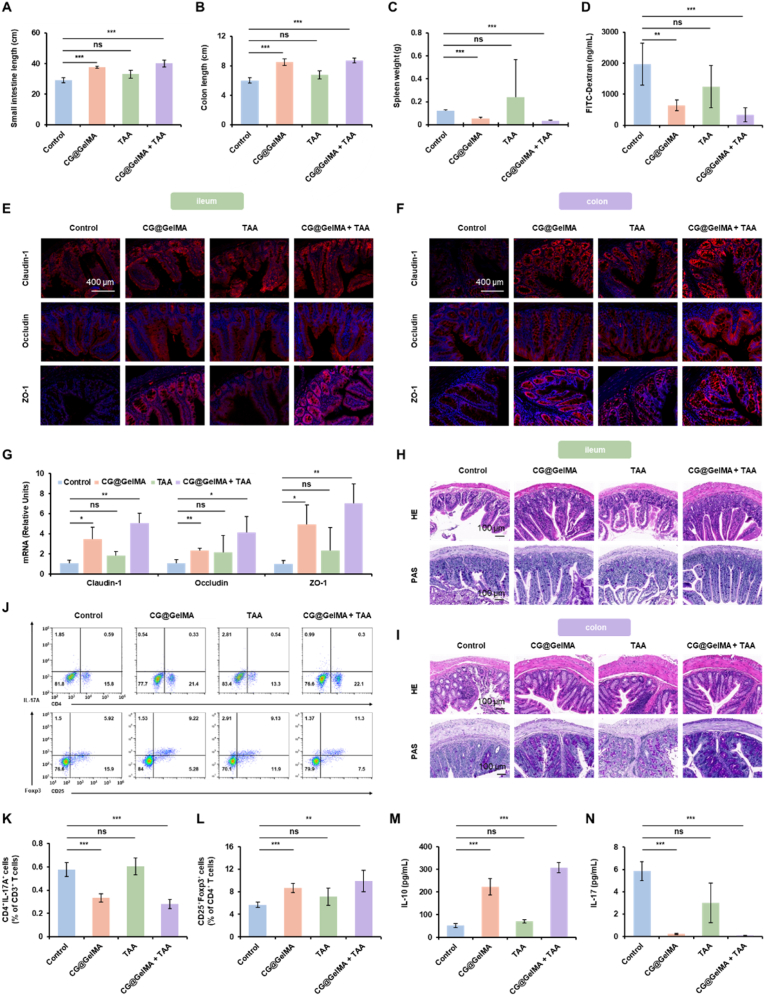
Effects of combined treatment on intestinal barrier integrity and systemic immunity. (A–B) Length of small intestine (A) and colon (B) of different treatment groups. (C) Spleen weight of different treatment groups. (D) Serum FITC-dextran concentration of different treatment groups. (E) Immunofluorescence staining of Claudin-1, Occludin, and ZO-1 in ileum tissues. Scale bars: 400 μm. (F) Immunofluorescence staining of Claudin-1, Occludin, and ZO-1 in colon tissues. Scale bars: 400 μm. (G) RT-qPCR analysis of Claudin-1, Occludin, and ZO-1 mRNA expression in colonic tissues of different treatment groups. (H–I) H&E and PAS staining of ileum (H) and colon (I). Scale bars: 100 μm. (J) Flow cytometry analysis of IL-17A^+^CD4^+^ T cells within the CD3^+^ T cell population and CD25^+^Foxp3^+^ regulatory T cells among CD4^+^ T cells in the spleen. (K–L) Quantification of Th17 (K) and Treg (L) populations of different treatment groups. (M − N) Serum levels of IL-10 (M) and IL-17 (N) of different treatment groups. Data are presented as means ± SD. Statistical significance: ∗*P* < 0.05, ∗∗*P* < 0.01, ∗∗∗*P* < 0.001.

Given the previously observed association between gut barrier dysfunction and arthritis severity, we next investigated whether treatment would lead to improvements in intestinal immune regulation and barrier integrity. Macroscopic examination revealed a notable shortening of both the small intestine and colon in the Control group, indicative of underlying intestinal inflammation or pathological changes. CG@GelMA group led to partial restoration of intestinal length, while the most pronounced improvement was observed in the CG@GelMA + TAA group (Fig. 6A and B and Fig. S27). The results of spleen weight measurement showed significant enlargement in the Control and TAA groups, whereas a marked reduction was observed in the CG@GelMA + TAA group. Notably, although TAA treatment effectively alleviated joint symptoms, it failed to improve intestinal abnormalities or reduce spleen enlargement, suggesting that CG@GelMA plays a critical role in regulating gut pathology and systemic immunity. (Fig. 6C).

Intestinal permeability was measured by the FITC-dextran assay. The Control group showed high serum FITC-dextran levels, indicating impaired gut barrier function. The CG@GelMA treatment significantly lowered intestinal permeability compared to Control, while TAA alone had little effect. Importantly, the CG@GelMA + TAA group showed the lowest FITC-dextran levels, indicating enhanced protection of the gut barrier (Fig. 6D). Immunofluorescence staining of the ileum and colon tissues demonstrated a marked reduction in the expression of tight junction proteins Claudin-1, Occludin, and ZO-1 in the Control group, indicating significant barrier disruption. Similarly weak fluorescence signals were observed in the TAA group, suggesting limited efficacy in restoring tight junction integrity. In contrast, the CG@GelMA group enhanced the expression of these proteins, with the highest signal intensity observed in the CG@GelMA + TAA group (Fig. 6E and F). Quantitative analysis confirmed significantly increased fluorescence intensities in both the CG@GelMA group and CG@GelMA + TAA group (Figs. S28–S29). In line with these findings, RT-qPCR analysis showed upregulated mRNA expression of Claudin-1, Occludin, and ZO-1 after treatment. Notably, both the CG@GelMA group and CG@GelMA + TAA group markedly enhanced the expression levels of these tight junction genes, with the combined treatment showing the strongest effect. By contrast, only a modest increase was observed in the TAA group compared with the Control (Fig. 6G).

Histological staining (H&E and PAS) revealed severe intestinal mucosal damage in the Control group, including villus atrophy, crypt disruption, and a marked loss of goblet cells. CG@GelMA treated group partially restored villus height and goblet cell numbers, while TAA group showed little improvement. Notably, the CG@GelMA + TAA group exhibited the most complete mucosal repair, with well-organized villi and crypts, restored goblet cells, and abundant mucin secretion (Fig. 6H and I and Fig.S30). To evaluate systemic immune responses, flow cytometry analysis of splenic lymphocytes showed that the CG@GelMA + TAA treatment significantly reduced the frequency of IL-17A^+^CD4^+^ T cells within the CD3^+^ T cell population compared to the Control treatment. Concurrently, the proportion of CD25^+^Foxp3^+^ regulatory T cells among CD4^+^ T cells was markedly increased (Fig. 6J–L). These cellular changes were also reflected in serum cytokine levels, with elevated IL-10 and decreased IL-17, indicating a shift toward a systemic anti-inflammatory profile (Fig. 6M and N). In summary, these findings suggest that CG@GelMA + TAA group not only alleviates joint inflammation but also restores gut barrier integrity, reduces systemic inflammation, and promotes immune homeostasis. This combined approach provides enhanced therapeutic benefits compared with the CG@GelMA group or TAA group alone, by simultaneously targeting both local joint pathology and gut-associated immune dysregulation.

### 3.8 Modulation of gut microbiota by combined oral and intra-articular treatment

**Fig. 7:**
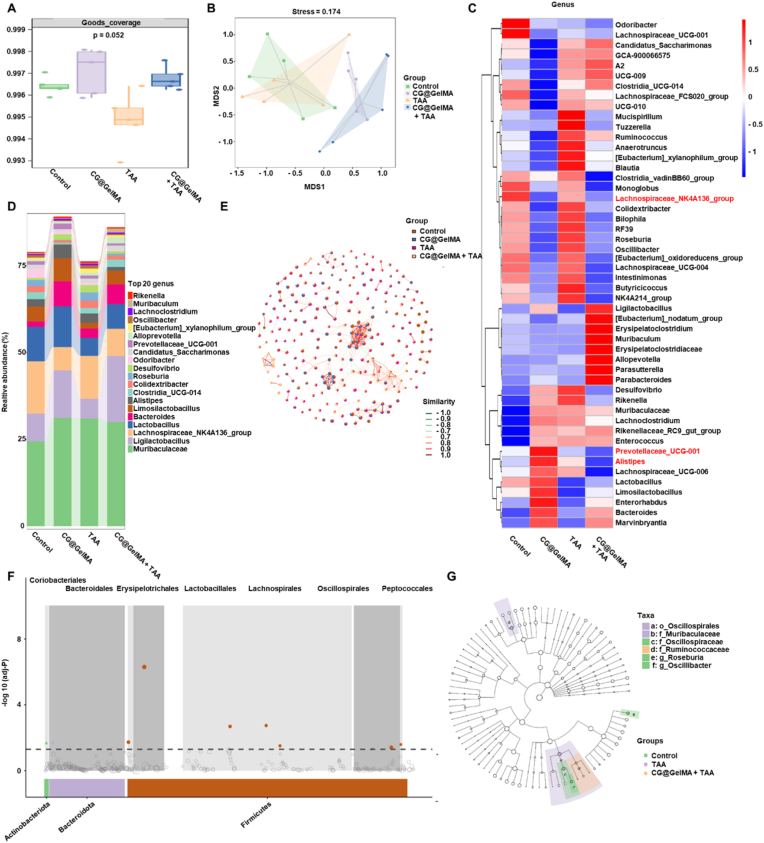
16S rDNA sequencing of gut microbiota in different treatment groups (A) Goods coverage of fecal microbiota in each group. (B) NMDS plot based on Bray–Curtis dissimilarity showing beta diversity among groups. (C) Heatmap of selected genera showing relative abundance across groups (genera associated with intestinal inflammation are highlighted in red, including *Prevotellaceae_UCG-001*, *Alistipes*, and *Lachnospiraceae_NK4A136_group*). (D) Bar plot of the top 20 genera in terms of relative abundance across samples. (E) Co-occurrence network of bacterial genera constructed using Spearman correlation. (F) Differentially abundant taxa at the order level identified by MetagenomeSeq analysis. (G) LEfSe analysis identifying discriminative taxa among treatment groups. Data are presented as means ± SD.

To explore the impact of combined CG@GelMA and TAA treatment on gut microbial composition, we performed 16S rDNA sequencing on fecal samples collected from each group. Goods coverage analysis confirmed that all groups achieved high sequencing depth, indicating reliable microbial community representation (Fig. 7A). To assess the functional potential of this restructured microbial community, we performed PICRUSt2-based prediction. The overall functional profile was dominated by metabolic pathways (Fig. S31), consistent with the core ecological roles of gut microbiota. Non-metric multidimensional scaling (NMDS) based on Bray–Curtis dissimilarity revealed a clear separation between the Control/TAA groups and the CG@GelMA - treated groups, with the CG@GelMA and CG@GelMA + TAA samples forming a distinct cluster. This pattern suggests that oral administration of CG@GelMA induces a substantial shift in gut microbial composition, while TAA alone has minimal impact on microbial structure (Fig. 7B).

At the genus level, the CG@GelMA + TAA group exhibited a marked increase in the relative abundance of *Ligilactobacillus*, a probiotic genus shown to enhance intestinal barrier function and exert anti-inflammatory effects. In contrast, several genera associated with intestinal inflammation and dysbiosis were significantly reduced after combined treatment. *Prevotellaceae_UCG-001*, reported to promote colonic inflammation via lithocholic acid metabolism, was notably decreased. *Alistipes*, linked to metabolic disorders and impaired gut-liver axis homeostasis, also showed reduced abundance. Likewise, *Lachnospiraceae_NK4A136_group*, previously associated with immune imbalance in rheumatoid arthritis, was significantly suppressed. These reductions suggest that the combined treatment alleviates microbial dysbiosis by increasing the abundance of beneficial taxa while decreasing inflammation-associated genera. These compositional changes were further supported by the heatmap and the top 20 genera bar plot, which showed elevated levels of *Ligilactobacillus* and reduced levels of *Prevotellaceae_UCG-001*, *Alistipes*, and *Lachnospiraceae_NK4A136_group*, reflecting a more balanced gut microbial community (Fig. 7C, D and Fig. S32).

Co-occurrence network analysis further revealed that certain genera from the CG@GelMA + TAA group exhibited strong positive correlations and a relatively concentrated distribution, indicating enhanced cooperative interactions under the combined intervention. In contrast, genera in other groups showed more scattered patterns and less organized associations (Fig. 7E). MetagenomeSeq analysis identified a significant enrichment of *Lactobacillales* and *Lachnospirales* in the combined group—both taxonomic orders known to contribute to the production of short-chain fatty acids (SCFAs), particularly acetate and butyrate, which support epithelial barrier integrity, immune modulation, and metabolic balance (Fig. 7F).Concurrently, targeted metabolomic analysis revealed that the concentrations of acetate, propionate, butyrate, isobutyrate, valerate, isovalerate, and hexanoate were all markedly increased in the CG@GelMA + TAA group compared to the Control (Fig. S33).

Further LEfSe analysis identified *Ruminococcaceae* as a representative genus specifically enriched in the combined treatment group (Fig. 7G). Members of this bacterial family are well-recognized for their ability to produce butyrate and other SCFAs, which support epithelial repair, decrease gut permeability, and modulate host inflammatory responses. Together, these results indicate that the combined administration of CG@GelMA and TAA not only alleviates joint pathology but also remodels the gut microbiota by enriching beneficial, SCFA-producing, and anti-inflammatory taxa, while concurrently reducing the abundance of bacteria associated with inflammatory dysbiosis. These microbiota alterations likely contribute critically to the systemic immunoregulatory and therapeutic effects observed in the CG@GelMA + TAA group.

### 3.9 FMT promotes the effectiveness of intra-articular therapy

**Fig. 8:**
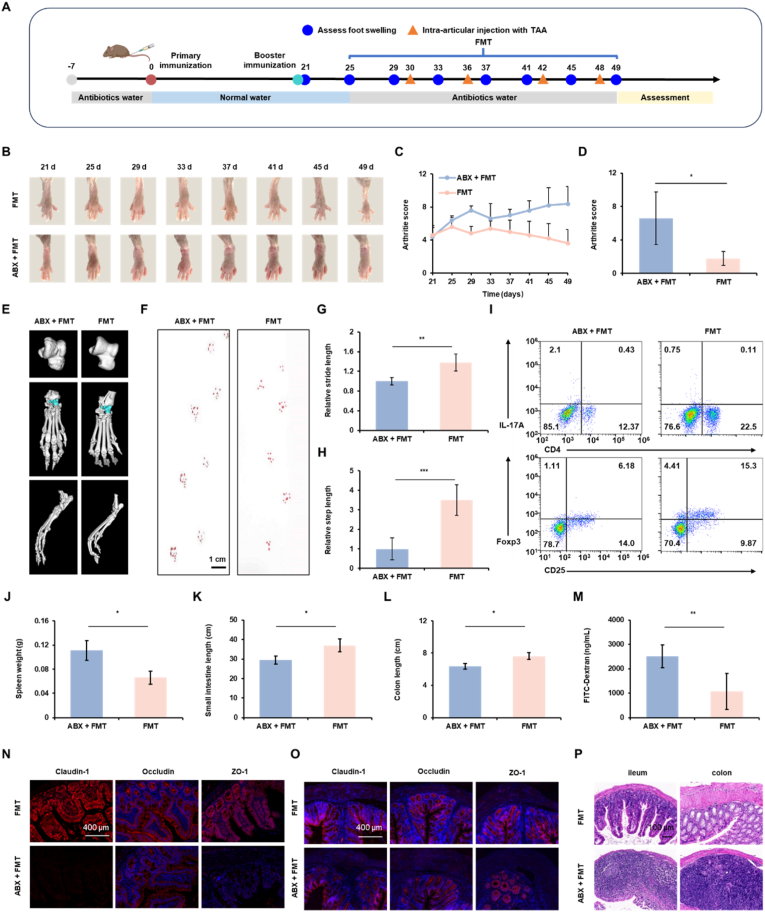
Fecal microbiota transplantation (FMT) from CG@GelMA - treated donors enhances the therapeutic effect of intra-articular TAA injection in CIA mice. (A) Schematic illustration of the experimental design involving FMT from CG@GelMA - treated donors, with or without concurrent antibiotic (ABX) administration. (B) Representative images of hind paws at multiple time points from day 21 to day 49. (C–D) Arthritis scores monitored over time (C) and quantified at day 49 (D). (E) Micro-CT images of hind limbs at day 49. (F–H) Gait assessment using paw print analysis (F), relative stride length (G), and relative step length (H). Scale bars: 1 cm. (I) Flow cytometric analysis of splenic IL-17A^+^CD4^+^ T cells (within CD3^+^ T cells) and CD25^+^Foxp3^+^ Tregs (within CD4^+^ T cells). (J–L) Spleen weight (J), small intestine length (K), and colon length (L) at day 49. (M) Serum FITC-dextran levels measured at day 49. (N–O) Immunofluorescence staining of Claudin-1, Occludin, and ZO-1 in the ileum (N) and colon (O) tissues. Scale bars: 400 μm. (P) Representative H&E staining images of ileum and colon tissues. Scale bars: 100 μm. Data are presented as means ± SD. Statistical significance: ∗*P* < 0.05, ∗∗*P* < 0.01, ∗∗∗*P* < 0.001.

To further investigate the contribution of gut microbiota restoration to the therapeutic efficacy of CG@GelMA, we established a fecal microbiota transplantation (FMT) model. Feces from CG@GelMA - treated mice were transplanted into CIA mice, with or without concurrent antibiotic (ABX) administration to disrupt microbial colonization (Fig. 8A). The ABX + FMT group received antibiotics from day −7 and throughout the FMT intervention to prevent stable microbial engraftment, while both groups were maintained on normal water from day 0–21 to avoid interference with arthritis induction. Daily FMT began on day 21, and intra-articular TAA injection was introduced from day 30.

Visual evaluation of hind paws from day 21 to day 49 showed that joint inflammation was more effectively resolved in the FMT group compared to the ABX + FMT group, with differences becoming more apparent toward the end of the intervention (Fig. 8B). Consistently, clinical arthritis scores displayed similar early fluctuations between groups but diverged later, with significantly higher scores in the ABX + FMT group by day 49 (Fig. 8C and D). Meanwhile, weight loss in the ABX + FMT group on day 49 indicated that disrupted microbial colonization adversely affected systemic metabolic homeostasis (Fig. S34). Micro-CT imaging showed more pronounced bone erosion in the ABX + FMT group, whereas the FMT group exhibited relatively preserved joint architecture. Furthermore, quantitative analysis revealed significantly reduced BMD, BV/TV, Tb.N, and Tb.Th in the ABX + FMT group compared to the FMT group (Fig. 8E and Fig. S35).

Locomotor analysis showed that the FMT group exhibited improved stride and step lengths compared to the ABX + FMT group, suggesting better recovery of joint function (Fig. 8F–H). Splenic flow cytometry revealed higher frequencies of CD4^+^IL-17A^+^ Th17 cells in the ABX + FMT group and elevated CD25^+^Foxp3^+^ Treg cells in the FMT group (Fig. 8I and Fig. S36), indicating differential modulation of immune subsets following FMT. Compared to the FMT group, mice in the ABX + FMT group exhibited notably increased spleen weight, suggesting pathological immune activation and systemic immune burden (Fig. 8J). In addition, both ileum and colonic lengths were shorter in the ABX + FMT group (Fig. 8K and L), consistent with aggravated inflammation and intestinal pathology. Serum FITC-dextran concentrations were significantly lower in the FMT group, indicating improved intestinal barrier integrity after microbiota restoration (Fig. 8M). Immunofluorescence staining of the ileum and colon sections showed higher expression of tight junction proteins Claudin-1, Occludin, and ZO-1 in the FMT group, whereas ABX + FMT treatment resulted in marked disruption of these signals (Fig. 8N–O and Fig. S37). Histological evaluation by H&E and PAS staining further demonstrated notable goblet cell depletion, mucosal damage, and crypt distortion in the ABX + FMT group, while the FMT group preserved a more intact epithelial architecture and mucus production (Fig. 8P, Fig. S38 and Fig. S39).

These findings suggest that regulating the gut microbiota is crucial for the therapeutic efficacy of CG@GelMA - based combination therapy, contributing to both local and systemic improvements in arthritis. It is worth noting that the use of antibiotics may directly alter host immunity and barrier function independently of microbiota. To this end, we conducted a supplementary FMT experiment using heat-inactivated microbiota. The results showed that this group exhibited a parallel phenotype to the ABX + FMT group, with neither achieving arthritis alleviation. This indicates that the therapeutic effect relies on viable bacteria, rather than other components of the fecal matter, thereby ruling out the possibility of direct immune damage by antibiotics (Figs. S40 and S41).

### 3.10 CG@GelMA - induced microbial restoration

**Fig. 9:**
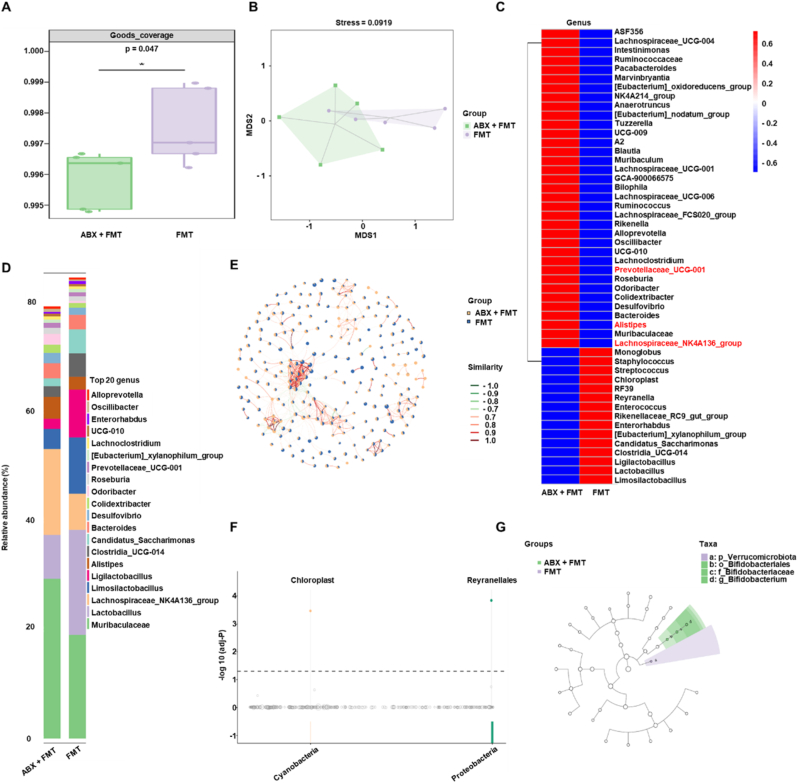
Analysis of gut microbiota differences between FMT and ABX + FMT groups. (A) Goods coverage of fecal microbiota in the FMT and ABX + FMT groups. (B) NMDS plot based on Bray–Curtis distance showing microbial community differences between the FMT and ABX + FMT groups. (C) Genus-level taxonomic composition of fecal microbiota in the two groups. (genera associated with intestinal inflammation are highlighted in red, including *Prevotellaceae_UCG-001*, *Alistipes*, and *Lachnospiraceae_NK4A136_group*) (D) Bar plot displaying the top 20 most abundant genera in fecal samples from the FMT and ABX + FMT groups. (E) Microbial co-occurrence network constructed from 16S rDNA sequencing data to visualize genus-level correlations between the two groups. (F) Differentially abundant taxa between FMT and ABX + FMT groups identified by MetagenomeSeq analysis. (G) Key discriminative microbial lineages between the two groups identified by LEfSe analysis.

To assess the microbial impact of fecal material derived from CG@GelMA - treated mice, 16S rDNA sequencing was performed on samples from the FMT and ABX + FMT groups. Sequencing coverage was high in both groups, ensuring adequate sampling depth (Fig. 9A). At the genus level, multiple taxa exhibited differential abundance between the FMT and ABX + FMT groups (Fig. 9B). Consistent with the microbial shifts observed in the CG@GelMA + TAA-treated mice, *Ligilactobacillus* was enriched in the FMT group, while genera previously associated with gut dysbiosis and inflammation-such as *Prevotellaceae_UCG-001*, *Alistipes*, and *Lachnospiraceae_NK4A136_group*—were reduced (Fig. 9C and Fig. S42). The relative abundance patterns of the top 20 genera (Fig. 9D) mirrored these changes, further supporting that feces derived from CG@GelMA - treated donors contributes to the restoration of a more favorable gut microbial community. The microbial co-occurrence network revealed that the FMT group exhibited higher network connectivity and modularity, indicating greater stability and functional cohesion within its microbial community (Fig. 9E). MetagenomeSeq analysis identified *Chloroplast* and *Reyranellales* as significantly enriched in the FMT group (Fig. 9F). The former likely reflects residual signals from transplanted CV-containing feces, given the chlorophyll-rich nature of *Chlorella vulgaris*. LEfSe analysis further identified *Verrucomicrobiota* as a key lineage enriched in the FMT group (Fig. 9G). Notably, *Akkermansia muciniphila*, a representative species of this phylum, has been shown in prior studies to exert protective effects by enhancing the intestinal mucus layer, improving tight junction integrity, and modulating immune responses, functions that play a crucial role in alleviating intestinal inflammation.

Collectively, these results demonstrate that oral CG@GelMA treatment induces distinct compositional and ecological alterations in the gut microbiota. This supports the critical role of microbiota modulation in mediating the therapeutic effects of CG@GelMA for arthritis attenuation.

### 3.11 Oral biosafety evaluation

To evaluate the potential toxicity of CG@GelMA following repeated oral administration, Balb/c mice were gavaged daily with PBS (control), CV, GPS, Blank@GelMA, or CG@GelMA for 30 consecutive days. At the end of the experiment, blood samples were collected for hematological and biochemical testing, and major organs were harvested for histological analysis. All groups showed blood and serum parameters within normal physiological ranges, without statistically significant differences compared to controls (Fig. S43A). Histological sections of the heart, liver, spleen, lungs, kidneys, stomach, and intestines revealed no pathological changes, such as tissue damage or inflammatory infiltration (Fig. S43B). These results indicate that CG@GelMA did not induce observable systemic or organ-specific toxicity under the tested conditions, supporting its safety for oral use in adjunctive treatment of rheumatoid arthritis and related systemic inflammatory disorders.

## 4 Conclusion

This study presents a synergistic therapeutic strategy for rheumatoid arthritis (RA) that combines oral CG@GelMA microspheres with intra-articular triamcinolone acetonide (TAA) injection. These thermoresponsive microspheres protect Chlorella vulgaris (CV) and ginseng polysaccharides (GPS) from gastric degradation and enable sustained intestinal release, thereby enhancing their bioavailability. *In vitro*, CG@GelMA restored gut barrier integrity by upregulating tight junction proteins (Claudin-1, Occludin, ZO-1), maintaining transepithelial electrical resistance, and limiting macromolecular permeability, while exerting potent antioxidative and anti-inflammatory effects.

In RA mice, oral CG@GelMA synergized with TAA to reduce joint inflammation and tissue damage, repair mucosal structure, restore intestinal barrier function, and modulate systemic immunity by decreasing pro-inflammatory IL-17A^+^CD4^+^ T cells and increasing regulatory T cells and IL-10 production. Analysis of the gut microbiota by 16S rDNA sequencing revealed significant reshaping of its composition. In parallel, targeted metabolomics revealed a marked increase in intestinal levels of several short-chain fatty acids (SCFAs). Fecal microbiota transplantation experiments confirmed that CG@GelMA reshaped the gut microbiota, enriching SCFA-producing and anti-inflammatory taxa while reducing dysbiosis-associated bacteria, thereby supporting gut–joint axis homeostasis. Although the specific CV-derived bioactive molecules that initiate this therapeutic cascade remain to be identified, future research employing untargeted metabolomics and compound isolation will pinpoint these upstream triggers.

These findings highlight thermoresponsive CG@GelMA microspheres as modulators of the gut–immune–joint axis in RA therapy, offering a promising strategy for RA and other systemic immune disorders associated with gut dysbiosis. As the specific CV-derived metabolites that link intestinal modulation to systemic and joint-level effects remain to be fully elucidated, future studies will focus on identifying and characterizing these key bioactive molecules and their precise mechanisms of action along the gut–blood–joint axis.

## CRediT authorship contribution statement

**Ruoxi Wang:** Writing – original draft, Formal analysis, Data curation, Conceptualization. **Aiying Tong:** Data curation. **Kangyu Jin:** Formal analysis, Data curation. **Runchang Yu:** Data curation. **Donghu Lin:** Formal analysis, Data curation. **Di Yang:** Formal analysis, Data curation. **Xiaoyang Liu:** Formal analysis, Data curation. **Jiarong Cui:** Formal analysis, Data curation. **Jiahua Niu:** Formal analysis, Data curation. **Yulin Cui:** Validation. **Haishuang Zhu:** Writing – review & editing, Project administration, Conceptualization. **Min Zhou:** Writing – review & editing, Supervision, Conceptualization.

## Ethics approval and consent to participate

All animal experiments were reviewed and approved by the Ethics Committee of Zhejiang University (approval number: ZJU20250848). All animal procedures were performed by the National Institute of Health Guide for the care and use of laboratory animals.

## Funding

This work was supported by the 10.13039/501100012166National Key R&D Program of China (2022YFA1105200), the Natural Science Foundation of Shandong Province (ZR2023ZD30), the Leading Innovative and Entrepreneur Team Introduction Program of Zhejiang (2022R01002), and the Key Research and Development Project of Zhejiang Province (2020C03035), we acknowledge the Core Facility of Zhejiang University School of Medicine for the technical support.

## Declaration of competing interest

The authors declare that they have no known competing financial interests or personal relationships that could have appeared to influence the work reported in this paper.
