# Supplementary material for: Harnessing the gut–immune–joint axis: Oral microalgae-based thermoresponsive microspheres enhance intra-articular therapy for rheumatoid arthritis
Source: Bioact Mater. 2026 Feb 10;61:72–91. doi: 10.1016/j.bioactmat.2026.01.037 (PMC12914198; doi:10.1016/j.bioactmat.2026.01.037)
Supplement: Multimedia component 1 [file mmc1.docx]

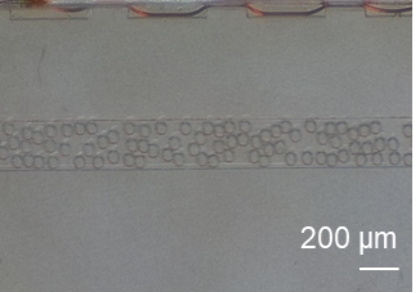


**Fig. S1.** Microscopic observation of microfluidic microsphere synthesis. Scale bars: 200 μm.


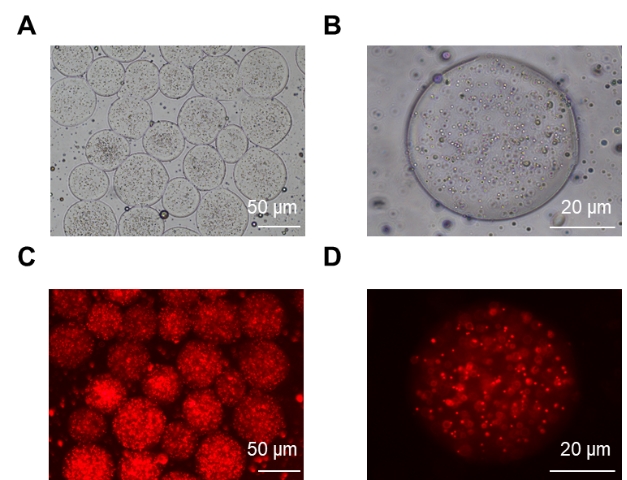


**Fig. S2.** Microscopic characterization of CG@GelMA.

(A, C) Low-magnification bright-field (A) and corresponding fluorescence images (C) of CG@GelMA (Scale bars: 50 μm).

(B, D) High-magnification bright-field (B) and fluorescence images (D) of CG@GelMA (Scale bars: 20 μm).


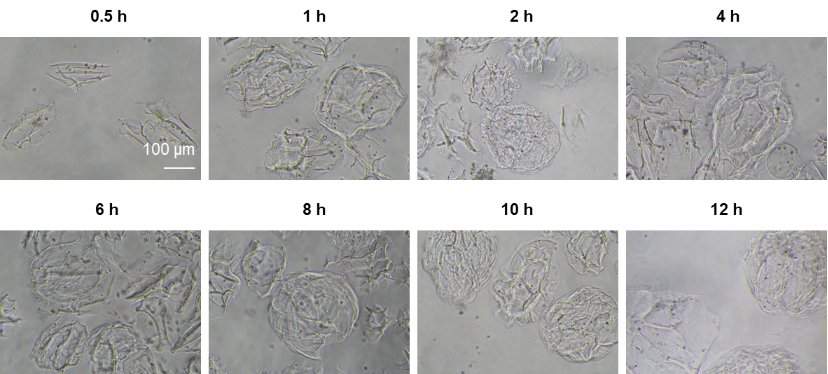


**Fig. S3**. Swelling behavior of lyophilized microspheres over time (Scale bars: 100 μm).


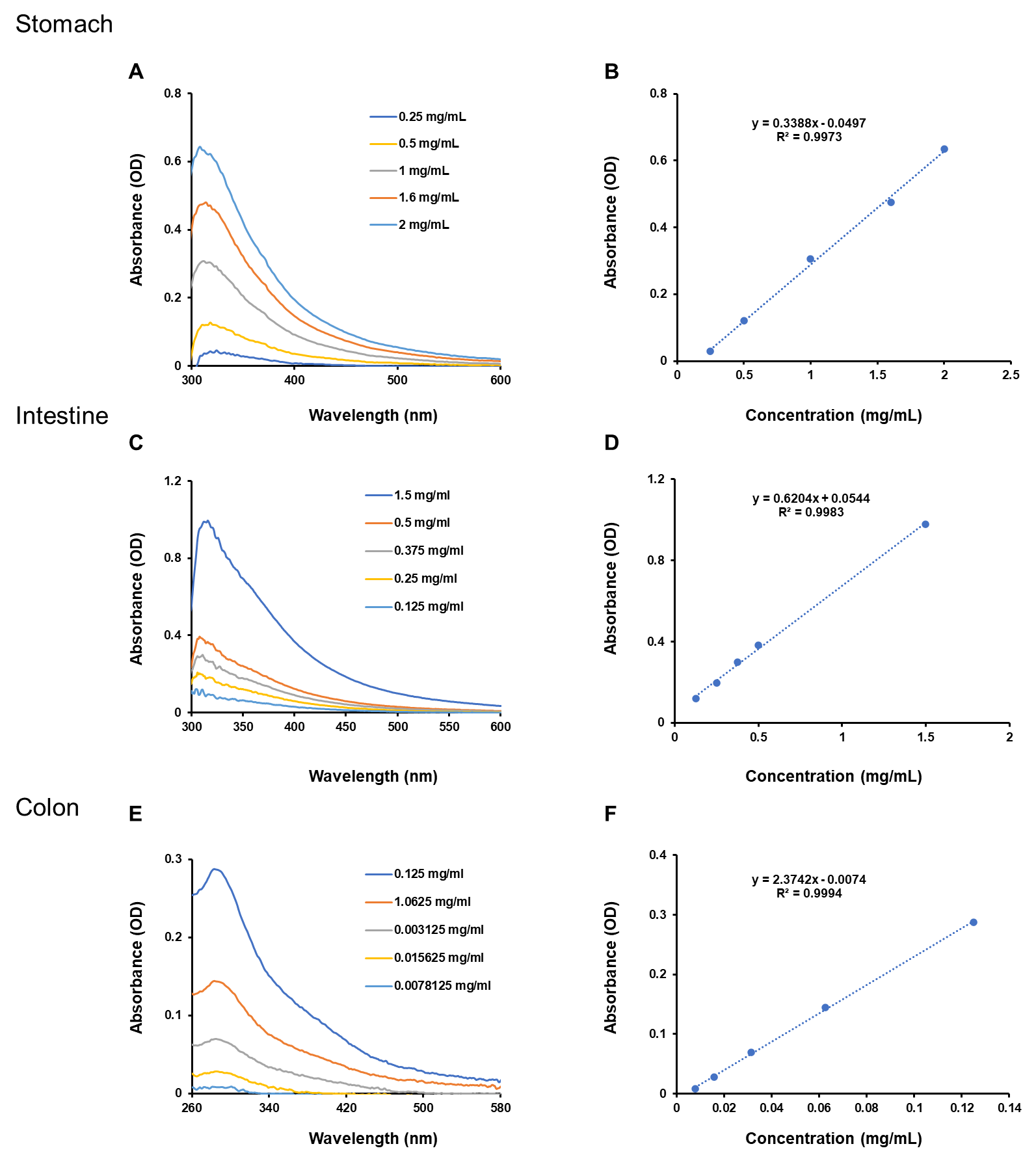


**Fig. S4**. UV-Vis-based standard curves of GPS constructed in simulated gastric fluid (SGF) (A–B), intestinal fluid (SIF) (C–D), and colonic fluid (SCF) (E–F).


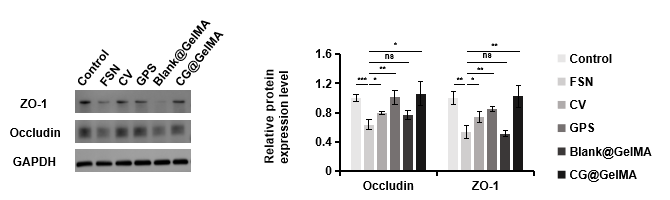


**Fig. S5**. Effects of different treatments on tight junction protein expression in FSN-induced Caco-2 cells. (A) Western blot analysis of tight junction. (B) Quantification of ZO-1 and Occludin expression levels in Caco-2 cells following different treatments. Data are presented as means ± SD. Statistical significance: **P* < 0.05, ***P* < 0.01, ****P* < 0.001.


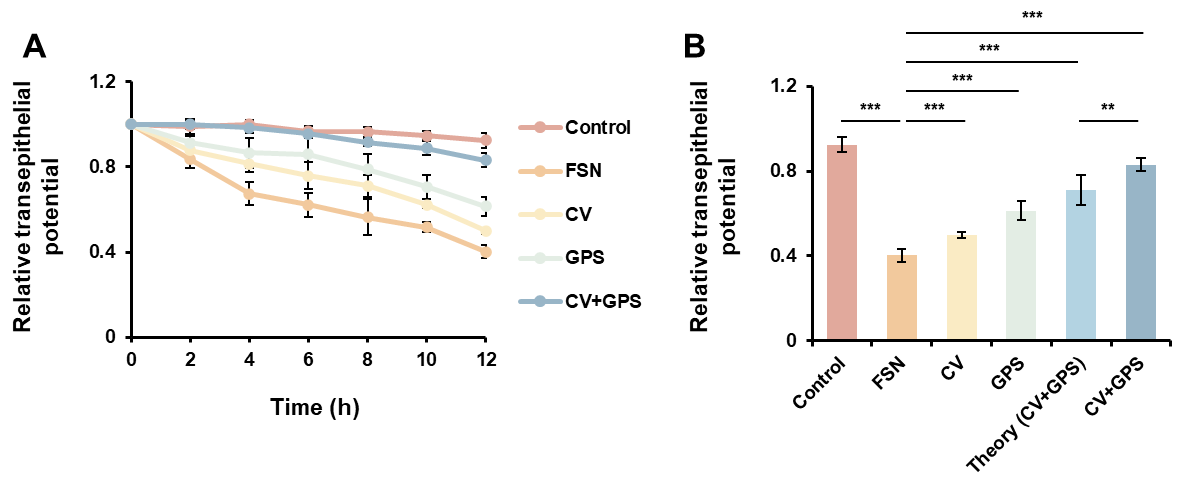


**Fig. S6**. Protective effects of different treatments on intestinal barrier integrity in an FSN-induced impairment model. (A) Relative transepithelial electrical resistance (TEER) measurements over time under different treatment conditions. (B) Comparison of relative TEER values across different treatment groups at the 12-hour time point. Data are presented as means ± SD. **P* < 0.05, ***P* < 0.01, and ****P* < 0.001.


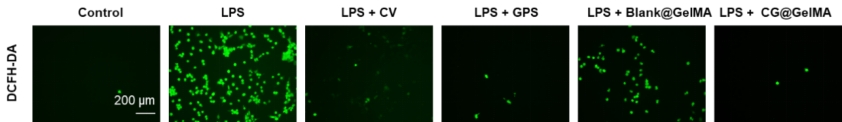


**Fig. S7**. Detection of ROS in IEC-6 cells via DCFH-DA staining (green fluorescence). Scale bar: 200 μm.


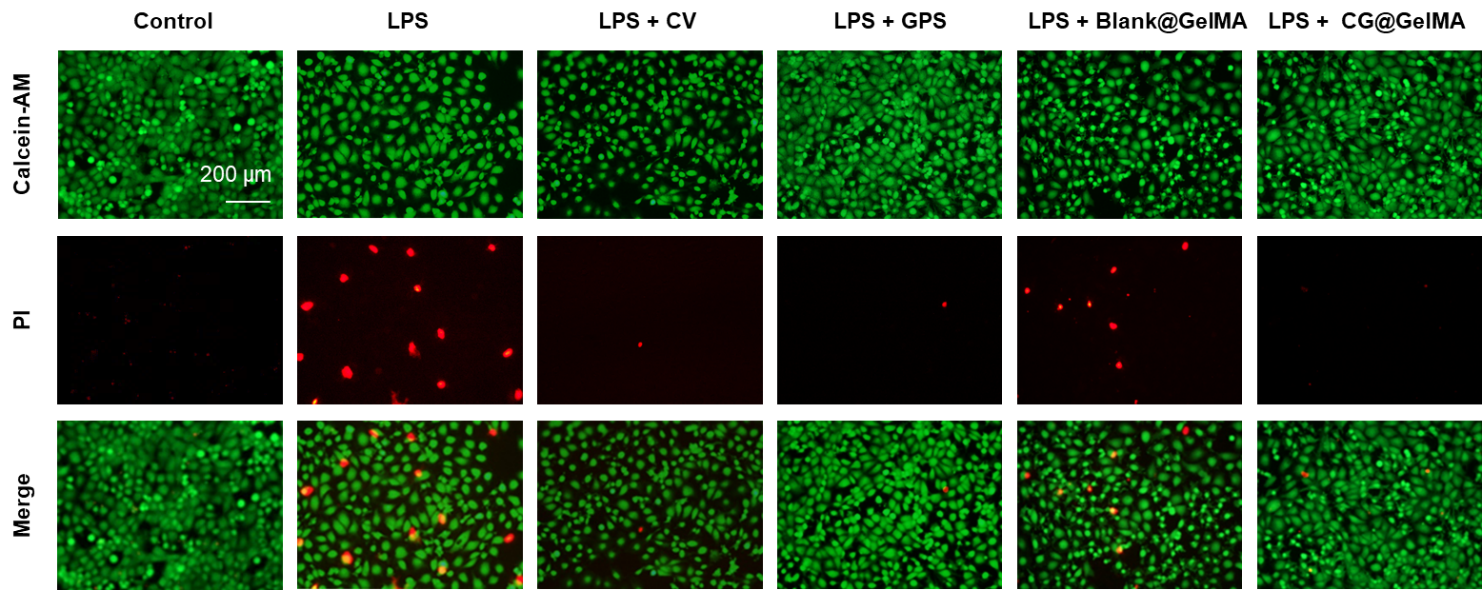


**Fig. S8**. Calcein-AM/PI staining of LPS-stimulated IEC-6 cells following treatment with CV, GPS, Blank@GelMA, or CG@GelMA. Scale bar: 200 μm.


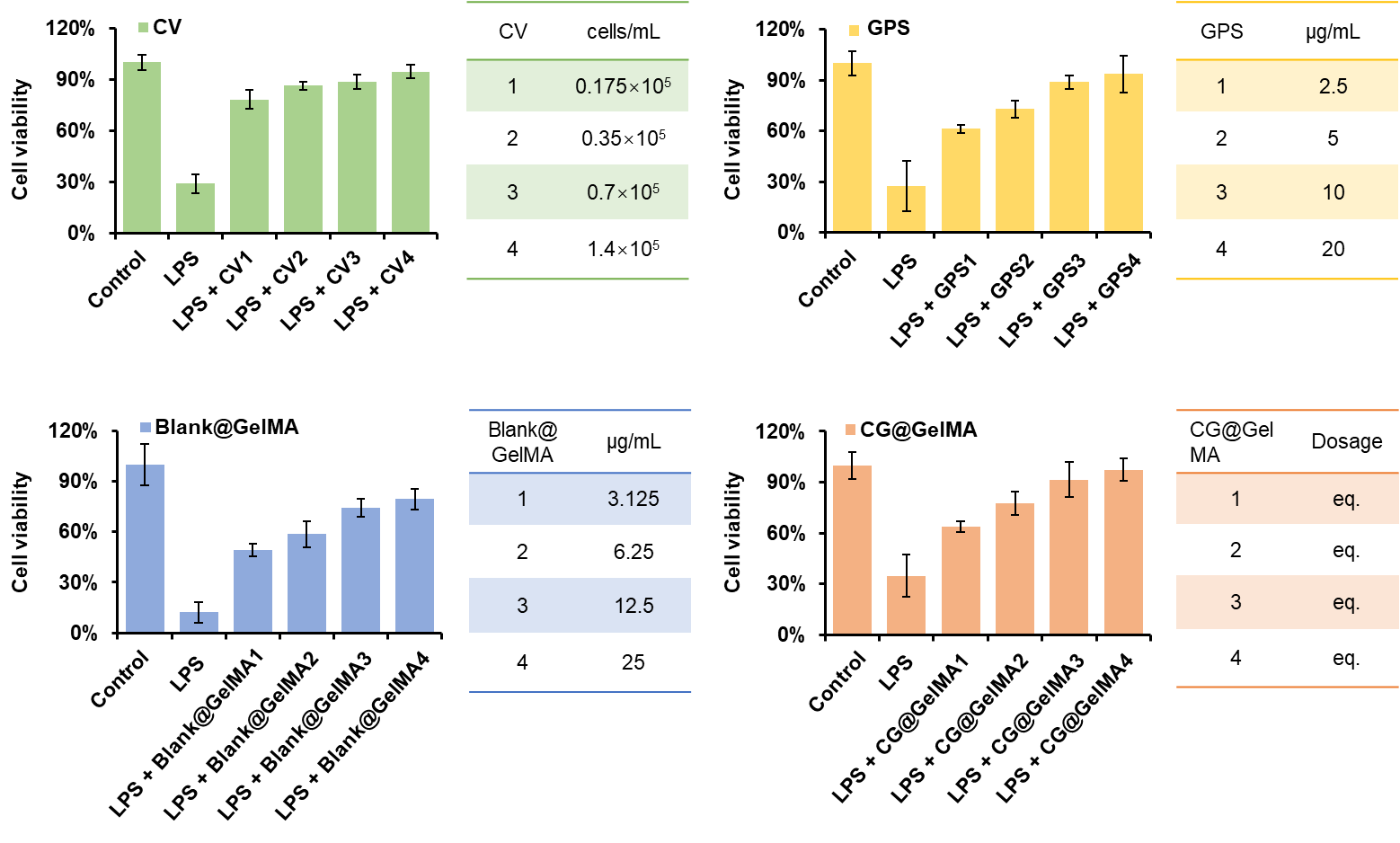


**Fig. S9**. Cell viabilities of LPS-stimulated IEC-6 cells after different treatments. Data are presented as means ± SD.


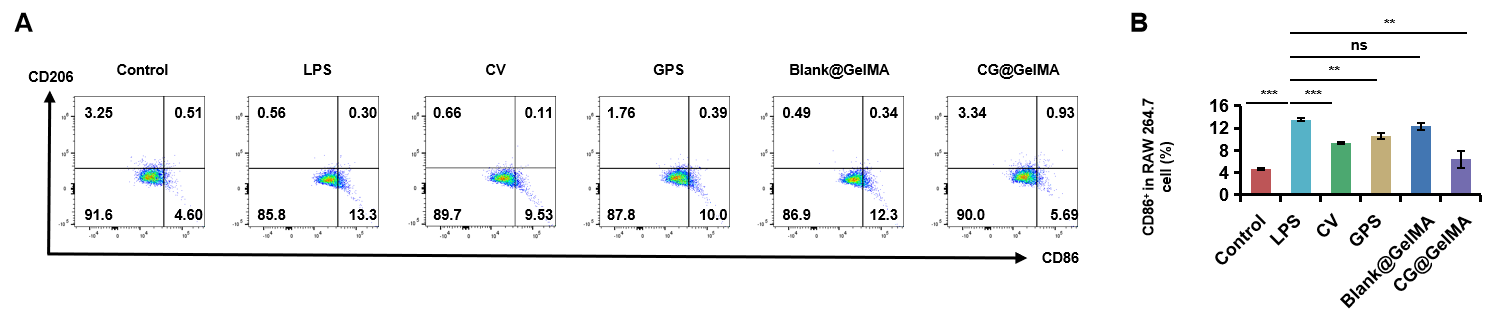


**Fig. S10**. Flow cytometric analysis of M1 macrophage polarization under different treatments. A. Representative flow cytometry plots showing the gating strategy for M1 macrophages (CD86⁺). B. Quantitative analysis of the percentage of M1 macrophages. Data are presented as means ± SD. **P* < 0.05, ***P* < 0.01, and ****P* < 0.001.


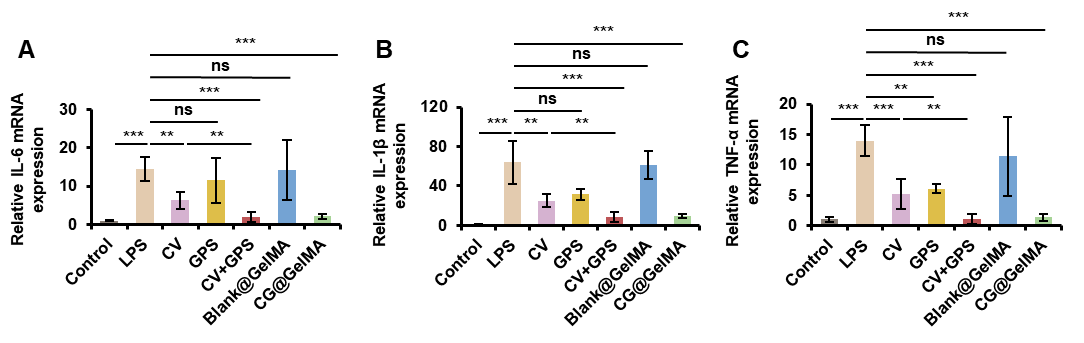


**Fig. S11**. Relative mRNA expression levels of IL-6, IL-1β and TNF-α in LPS-stimulated IEC-6 cells treated with different treatments. Data are presented as means ± SD. **P* < 0.05, ***P* < 0.01, and ****P* < 0.001.


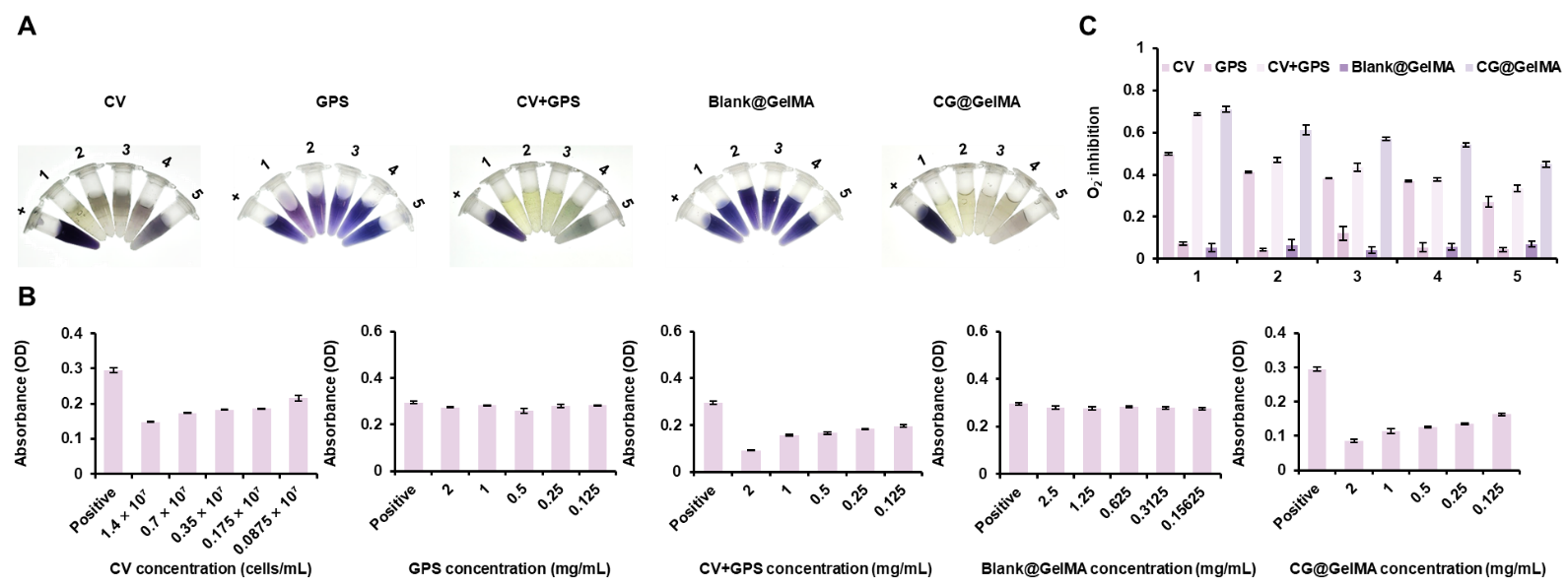


**Fig. S12**. Scavenging of superoxide anions (O₂·⁻) by CV, GPS, CV+GPS, Blank@GelMA and CG@GelMA.

(A) Representative photographs showing the color changes of the reaction products.

(B) The absorbance of the reaction products at 560 nm.

(C) The O₂·⁻ scavenging activity, expressed as inhibition rate. Numbers 1 to 5 represent samples from high to low concentrations, respectively. For the CV+GPS and CG@GelMA groups, CV and GPS were applied as a paired gradient at a fixed ratio. The abscissa indicates the GPS concentration gradient (mg/mL). Data are presented as means ± SD.

**
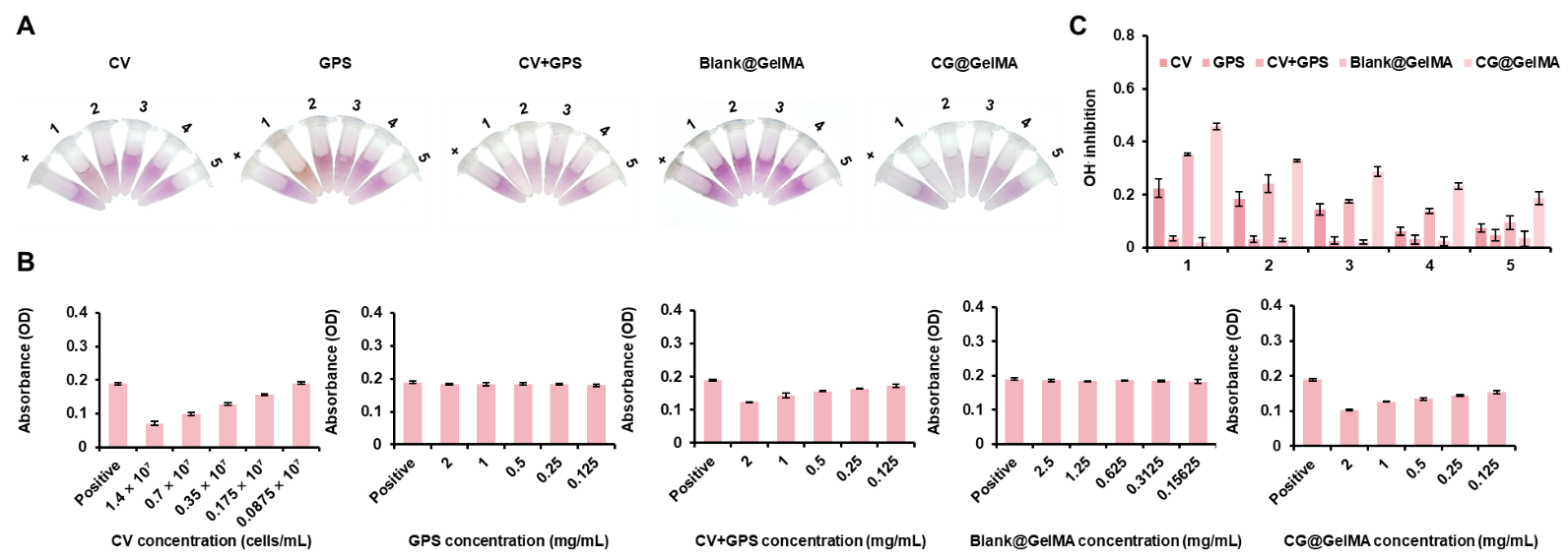
**

**Fig. S13**.Scavenging of hydroxyl radical (·OH) by CV, GPS, CV+GPS, Blank@GelMA and CG@GelMA.

(A) Representative photographs showing the color changes of the reaction products.

(B) The absorbance of the reaction products at 510 nm.

(C) The ·OH scavenging activity, expressed as inhibition rate. Numbers 1 to 5 represent samples from high to low concentrations, respectively. For the CV+GPS and CG@GelMA groups, CV and GPS were applied as a paired gradient at a fixed ratio. The abscissa indicates the GPS concentration gradient (mg/mL). Data are presented as means ± SD.


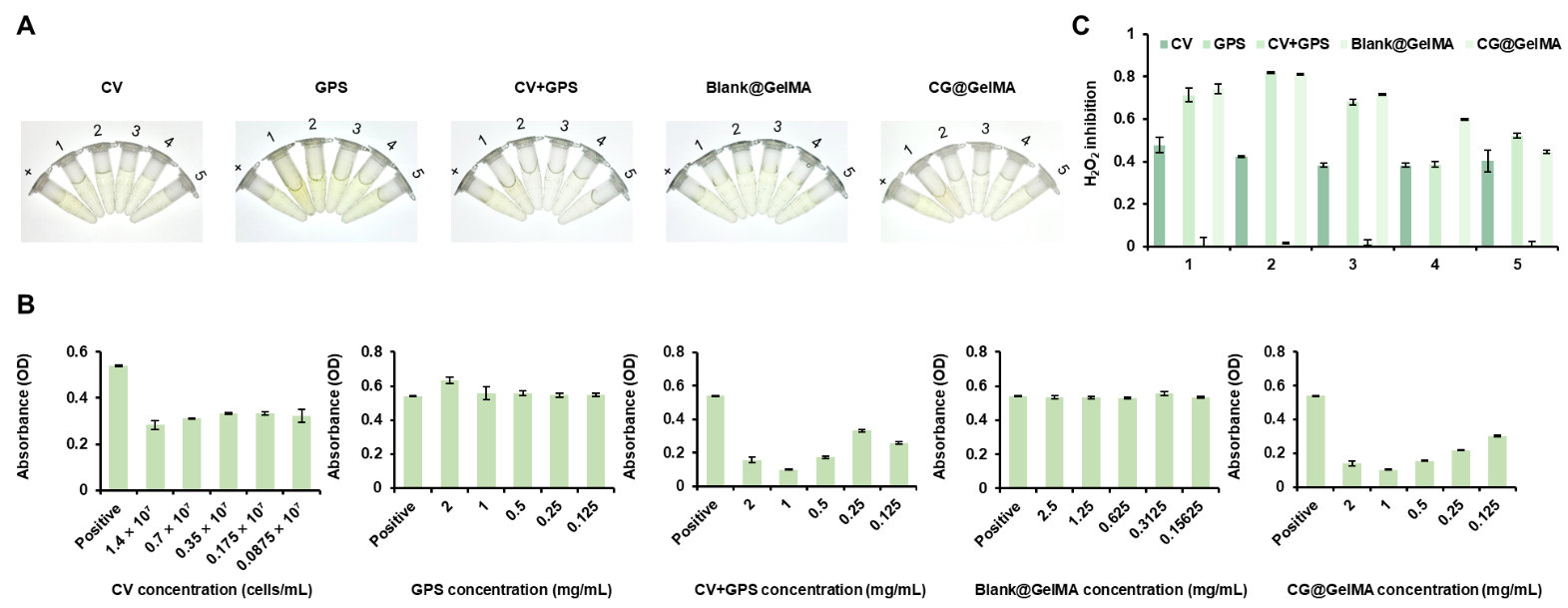


**Fig. S14**.Scavenging of hydrogen peroxide (H_2_O_2_) by CV, GPS, CV+GPS, Blank@GelMA and CG@GelMA.

(A) Representative photographs showing the color changes of the reaction products.

(B) The absorbance of the reaction products at 405 nm.

(C) The H_2_O_2_ scavenging activity, expressed as inhibition rate. Numbers 1 to 5 represent samples from high to low concentrations, respectively. For the CV+GPS and CG@GelMA groups, CV and GPS were applied as a paired gradient at a fixed ratio. The abscissa indicates the GPS concentration gradient (mg/mL). Data are presented as means ± SD.


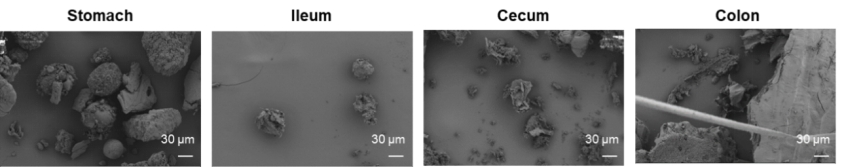


**Fig. S15**. SEM characterization of contents from stomach, ileum, cecum, and colon after oral administration of CG@GelMA. Scale bar: 30 μm.


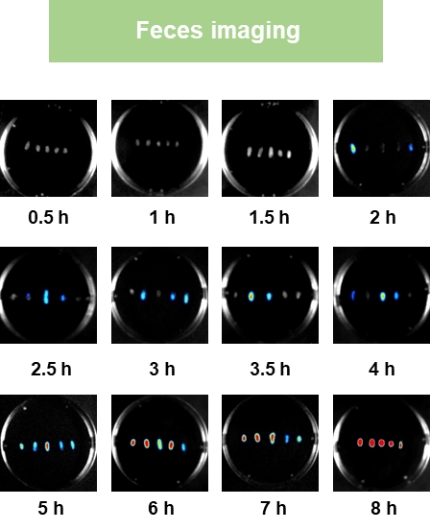


**Fig. S16.** Fecal fluorescence imaging over time following oral administration of CG@GelMA, detected via chlorophyll autofluorescence.


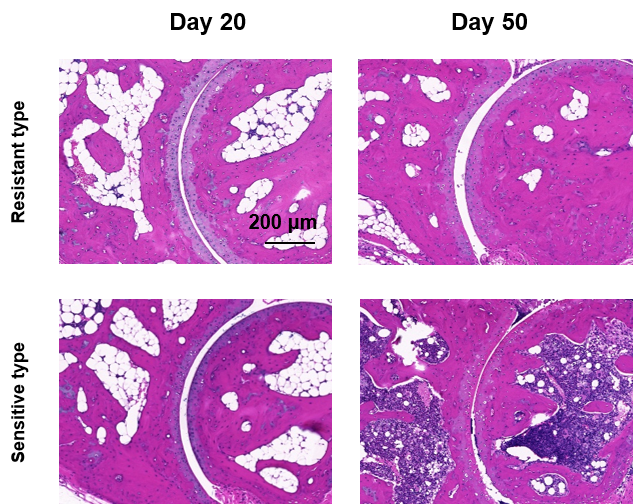


**Fig. S17.** Histological assessment of joint tissues stained with H&E in resistant and sensitive types at day 20 and day 50 post-treatment. Scale bar: 200 μm.


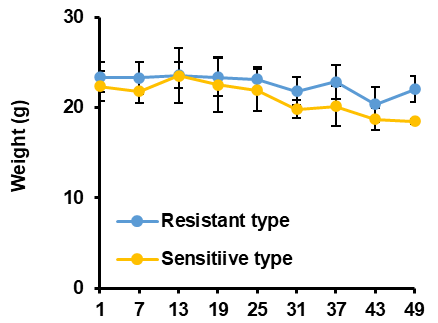


**Fig. S18.** Body weight changes in sensitive type and resistant type mice throughout the experimental period. Data are presented as means ± SD.


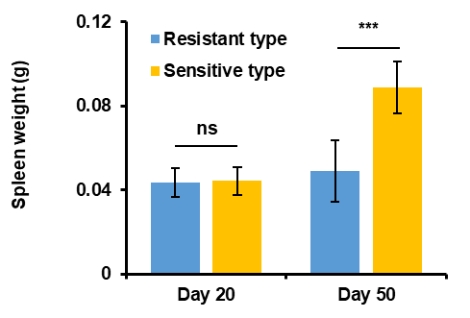


**Fig. S19.** Spleen weights in sensitive type and resistant type mice measured on day 20 and day 50. Data are presented as means ± SD. **P* < 0.05, ***P* < 0.01, and ****P* < 0.001.


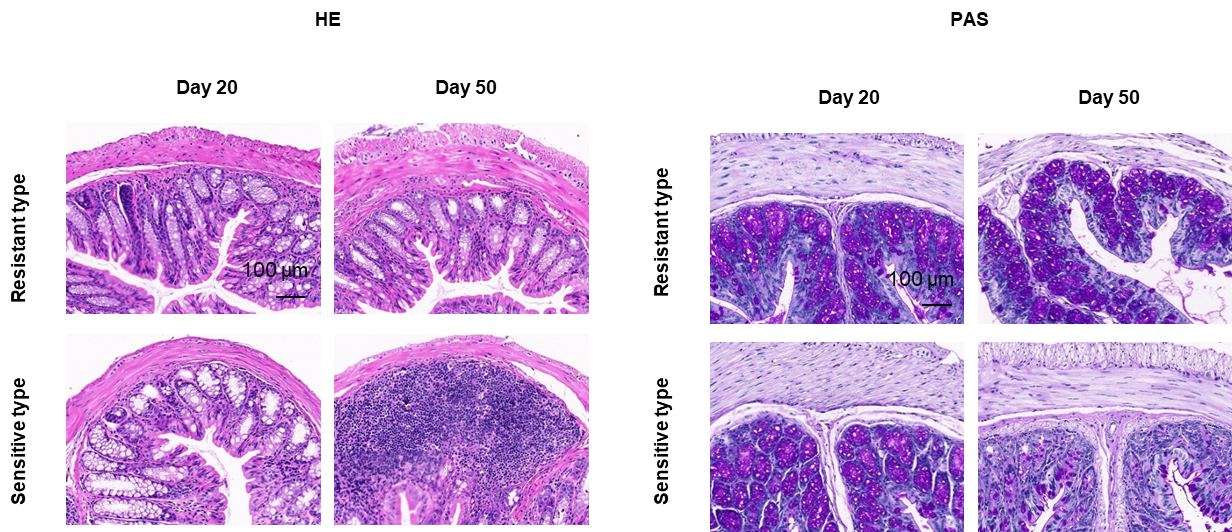


**Fig. S20.** Representative H&E (left) and PAS (right) staining of colon tissues from sensitive type and resistant type mice on day 20 and day 50. Scale bars: 100 µm.


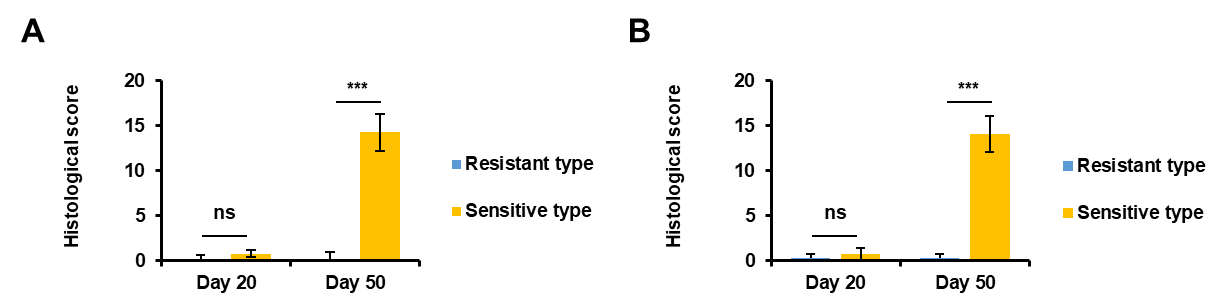


**Fig. S21.** Histological score of ileum (A) and colon (B). Data are presented as means ± SD. Statistical significance: **P* < 0.05, ***P* < 0.01, ****P* < 0.001.


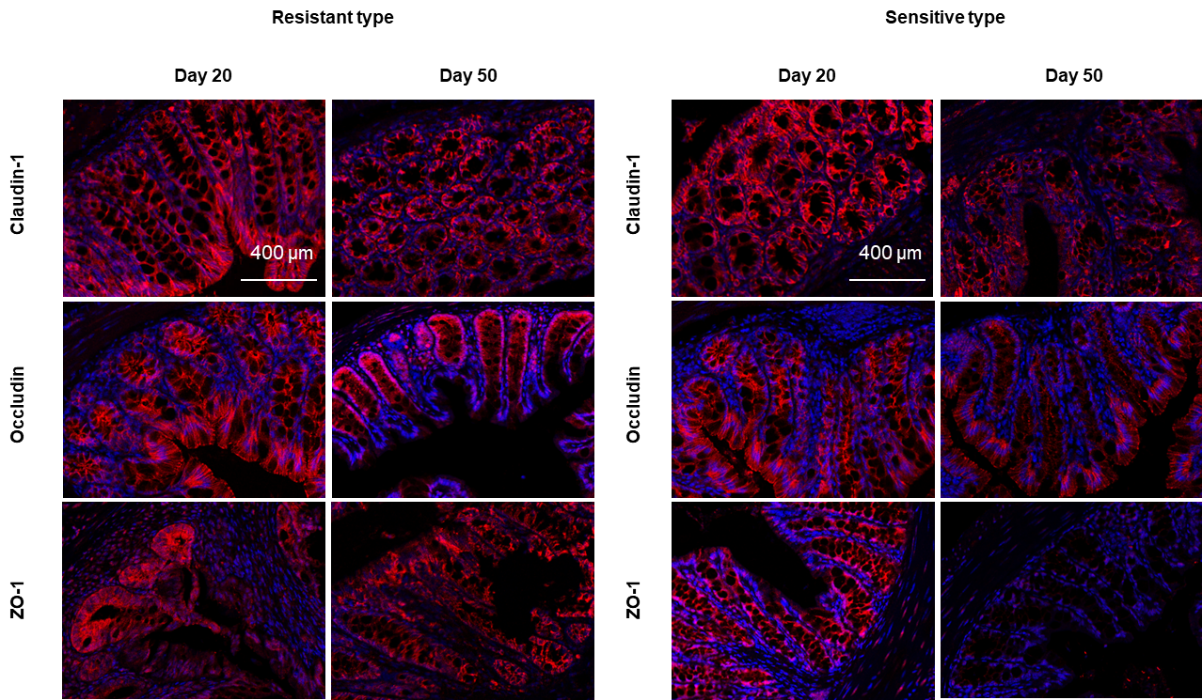


**Fig. S22**. Immunofluorescence staining of Claudin-1, Occludin, and ZO-1 in colonic tissues from sensitive type and resistant type mice on day 20 and day 50. Scale bars: 400 µm.


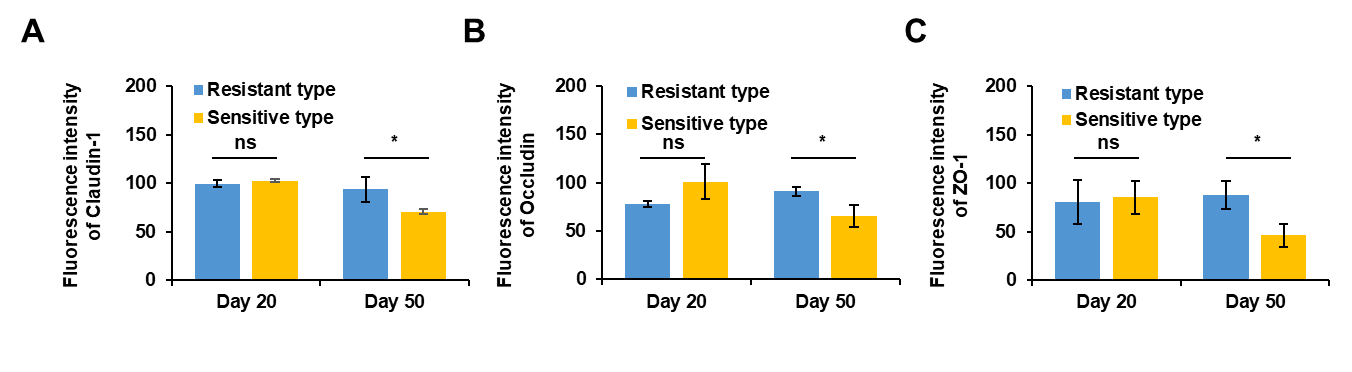


**Fig. S23.** Quantitative analysis of fluorescence intensity for Claudin-1 (A), Occludin (B), and ZO-1 (C) in colonic sections from sensitive type and resistant type mice on day 20 and day 50. Data are presented as means ± SD. Statistical significance: **P* < 0.05, ***P* < 0.01, ****P* < 0.001.


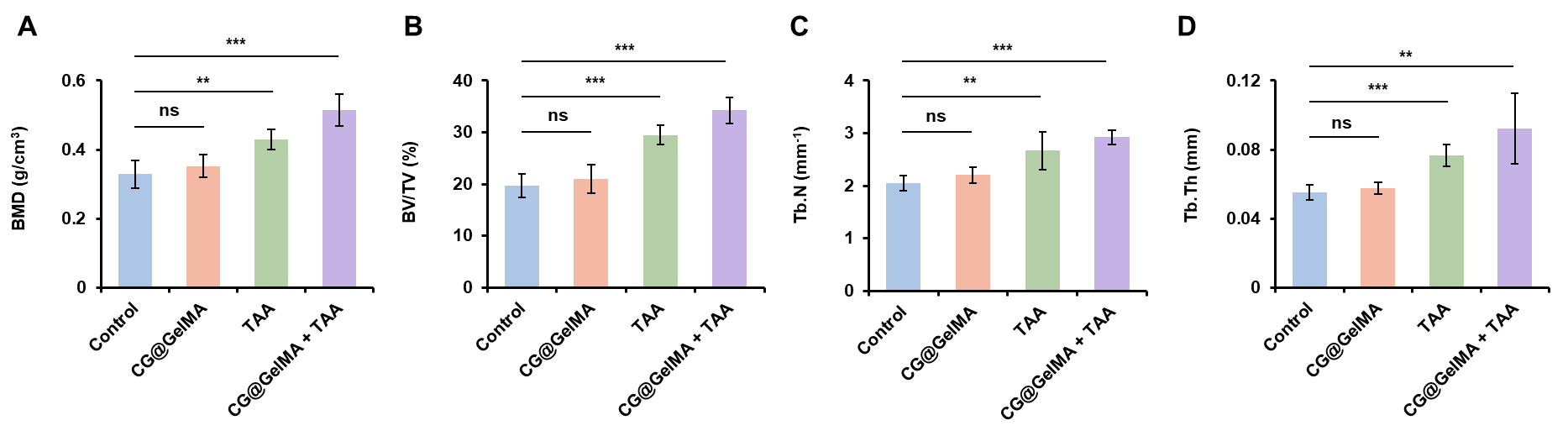


**Fig. S24.** Quantitative micro-CT analysis of bone mass and microarchitecture (BMD, BV/TV, Tb.N, Tb.Th) in various treatment groups. Data are presented as means ± SD. Statistical significance: **P* < 0.05, ***P* < 0.01, ****P* < 0.001.


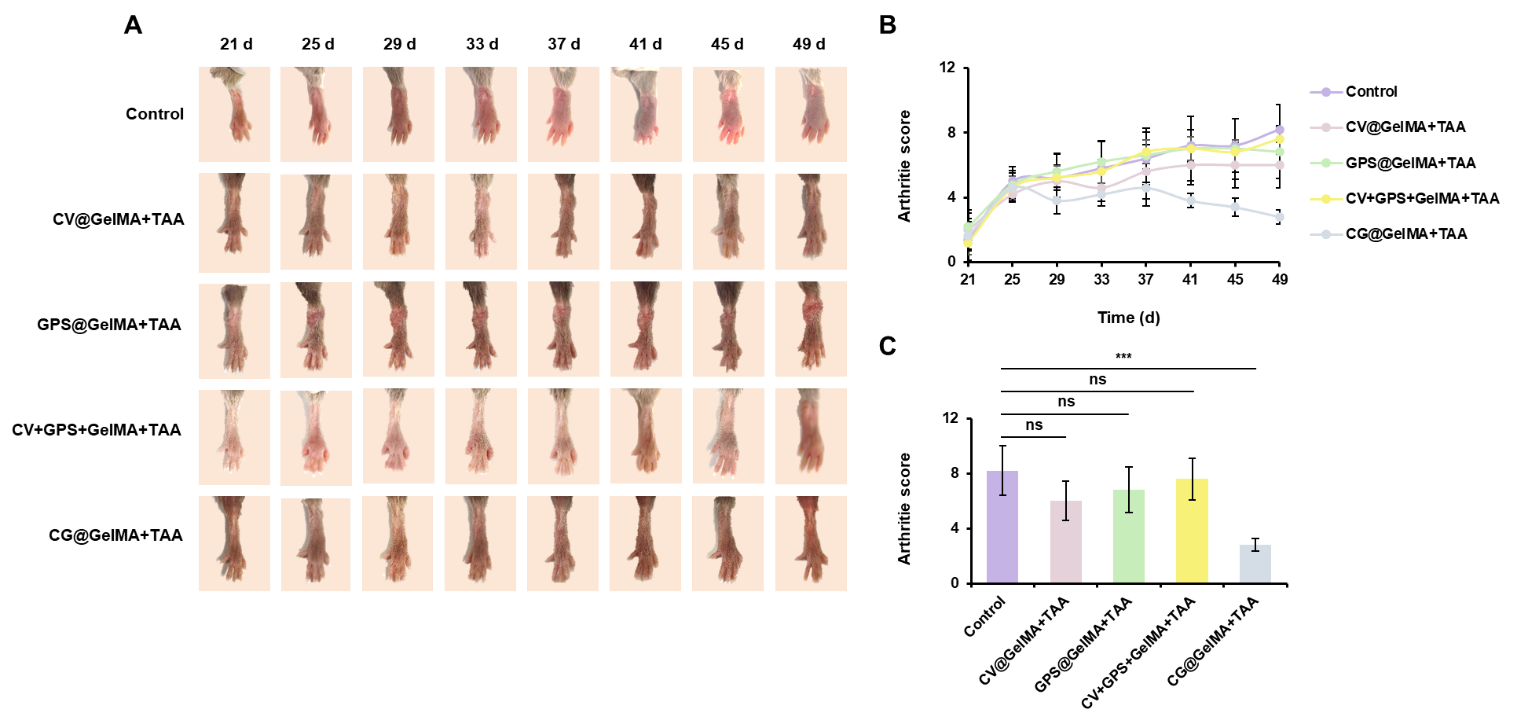


**Fig. S25**. Comparative analysis of different treatments (A) Representative joint morphology in different groups. (B–C) Arthritis scores recorded throughout the study (B) and on day 49 (C) in different groups. Data are presented as means ± SD. Statistical significance: **P* < 0.05, ***P* < 0.01, ****P* < 0.001.


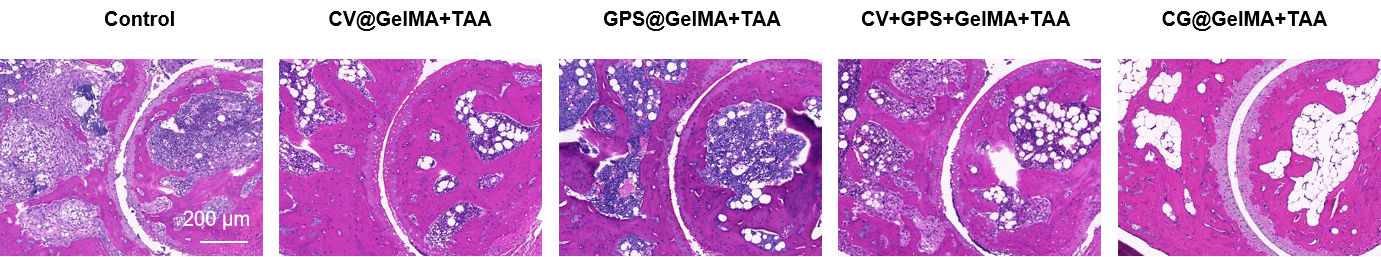


**Fig.S26**. Histological assessment of joint tissues stained with H&E in different treatments. Scale bars: 200 µm.


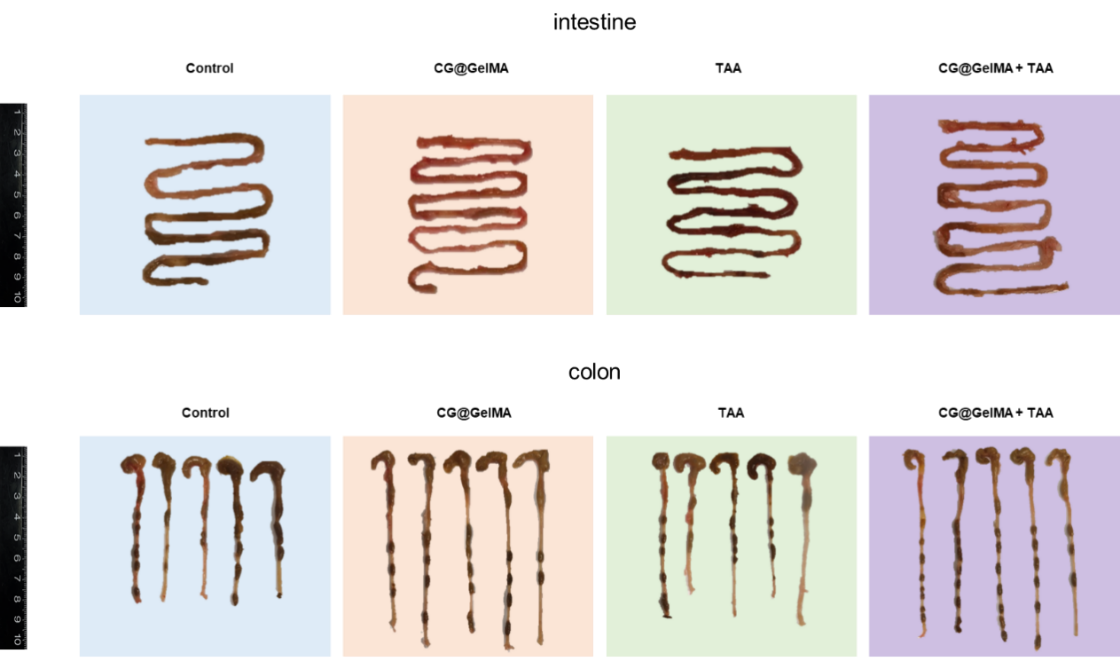


**Fig. S27**. Representative photographs of the intestine and colon from different treatment groups.


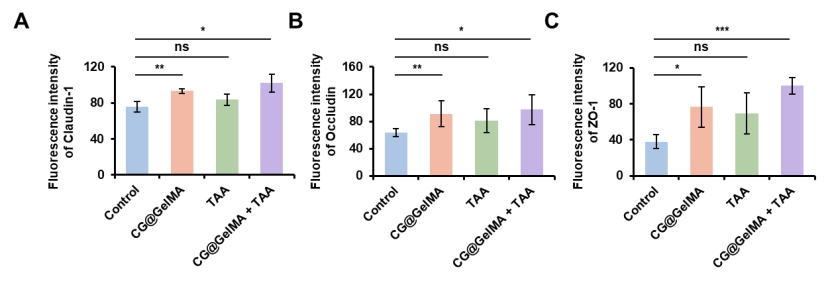


**Fig. S28.** Quantitative analysis of fluorescence intensity for Claudin-1 (A), Occludin (B), and ZO-1 (C) in ileum tissues in different treatments. Data are presented as means ± SD. Statistical significance: **P* < 0.05, ***P* < 0.01, ****P* < 0.001.


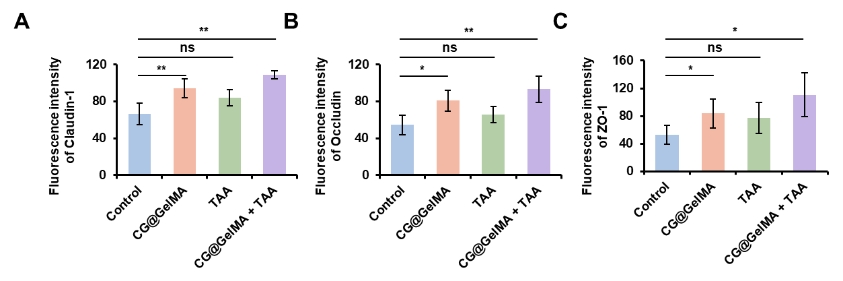


**Fig. S29.** Quantitative analysis of fluorescence intensity for Claudin-1 (A), Occludin (B), and ZO-1 (C) in colonic tissues in different treatments. Data are presented as means ± SD. Statistical significance: **P* < 0.05, ***P* < 0.01, ****P* < 0.001.


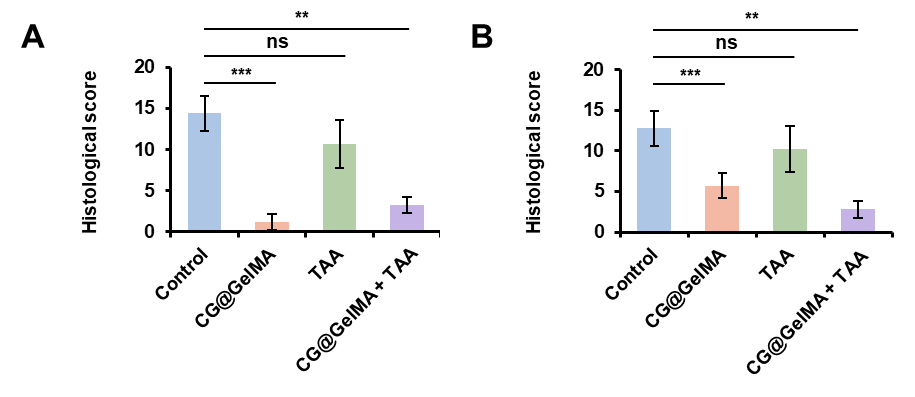


**Fig.S30**. Histological score of ileum (A) and colon (B). Data are presented as means ± SD. Statistical significance: **P* < 0.05, ***P* < 0.01, ****P* < 0.001.


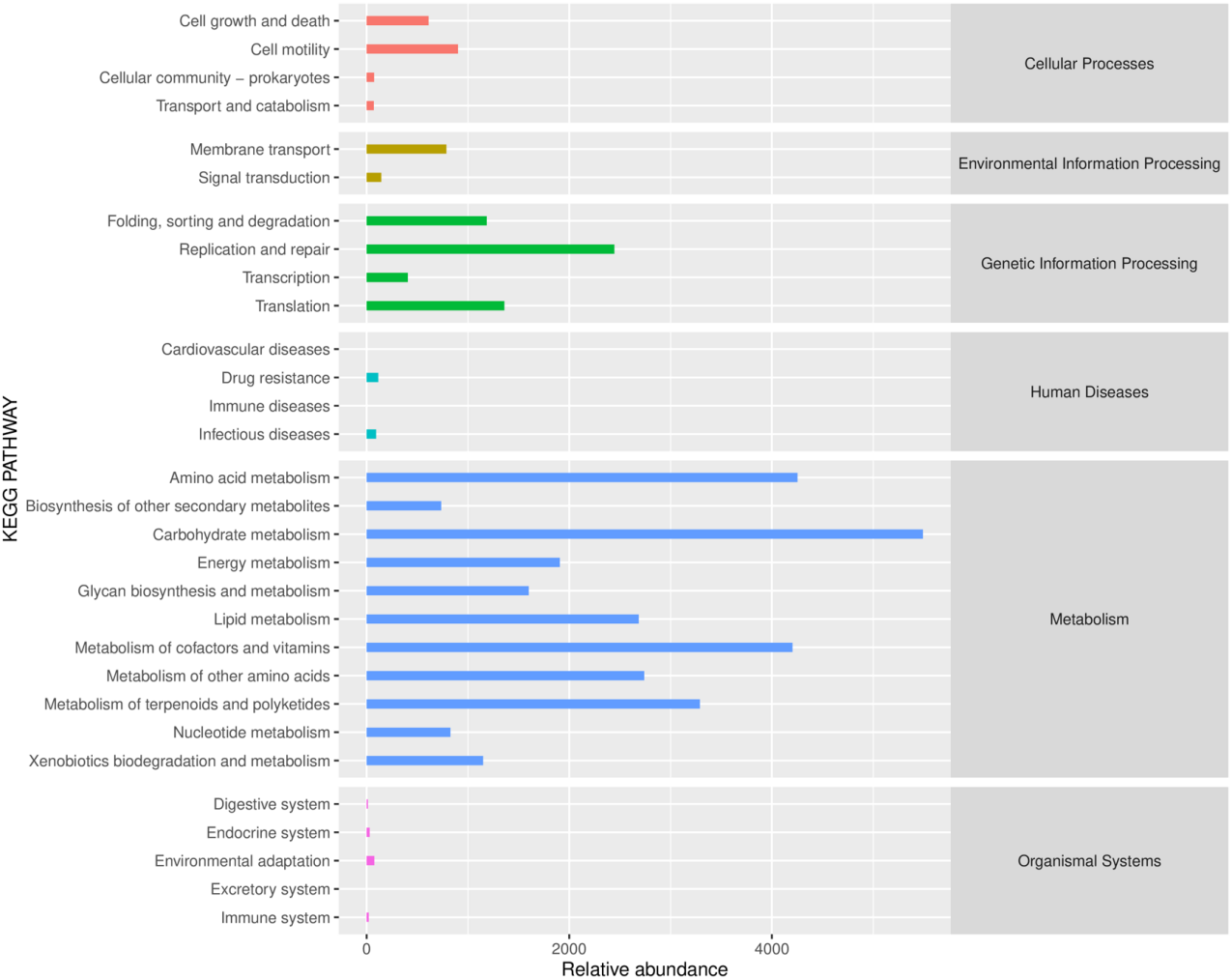


**Fig. S31.** Predicted relative abundance profile of KEGG functional pathways in gut microbiota based on PICRUSt2.


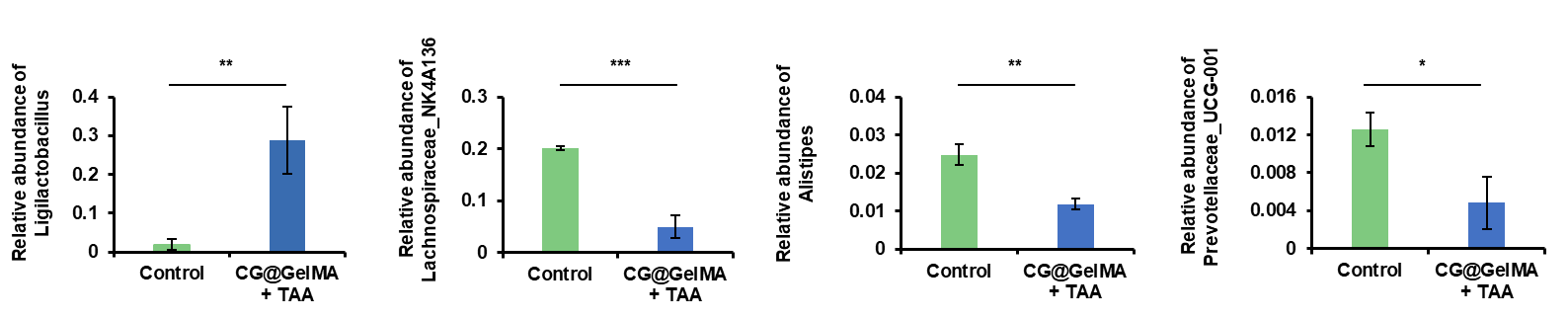


**Fig. S32.** Relative abundance of *Ligilactobacillus*, *Lachnospiraceae_NK4A136*, *Alistipes* and *Prevotellaceae_UCG-001.* Data are presented as means ± SD. Statistical significance: **P* < 0.05, ***P* < 0.01, ****P* < 0.001.


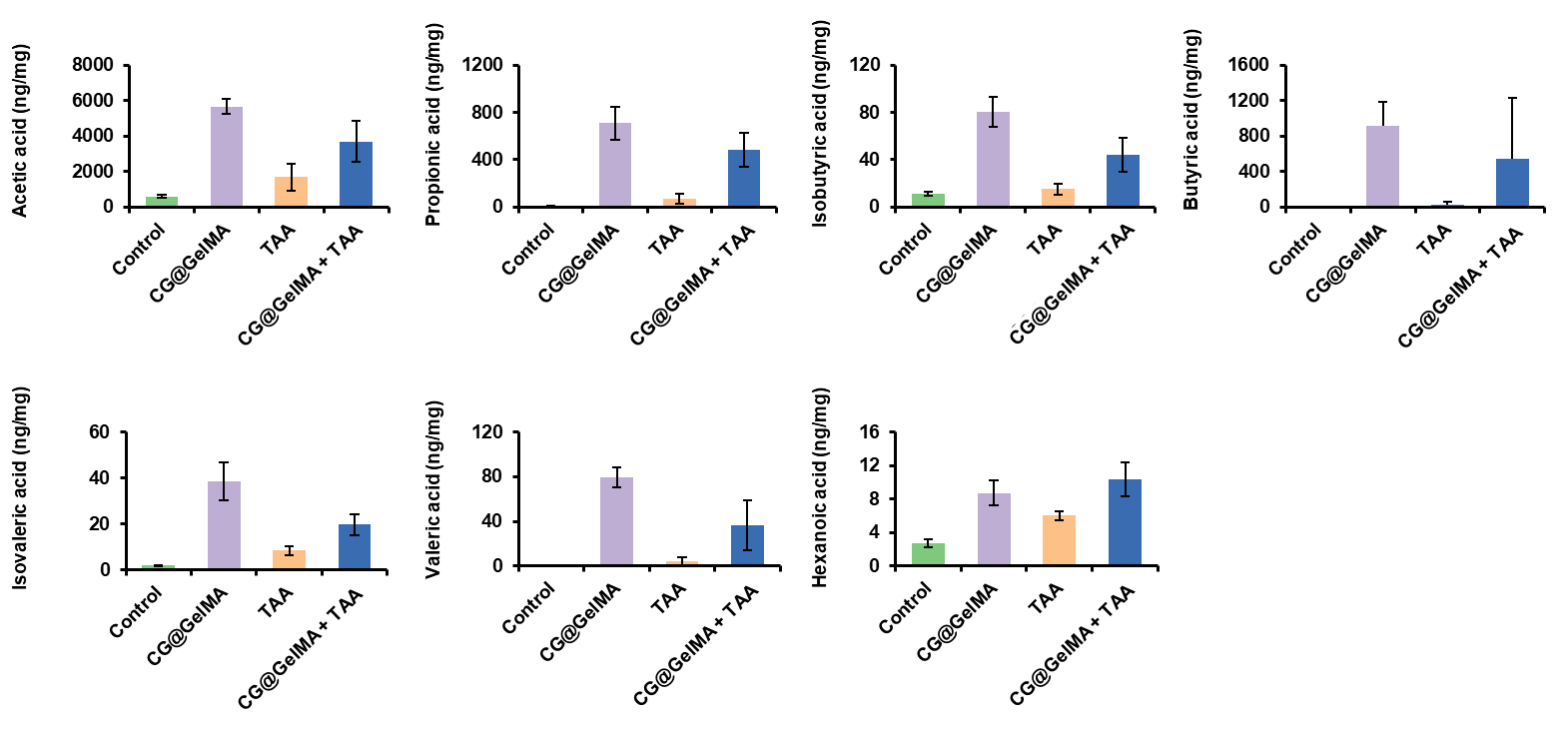


**Fig. S33.** Quantitative analysis of short-chain fatty acids (SCFAs) in feces among different treatment groups. Data are presented as means ± SD.


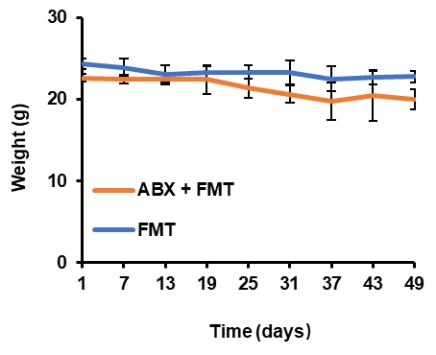


**Fig.S34.** Body weight changes in different treatments throughout the experimental period. Data are presented as means ± SD.


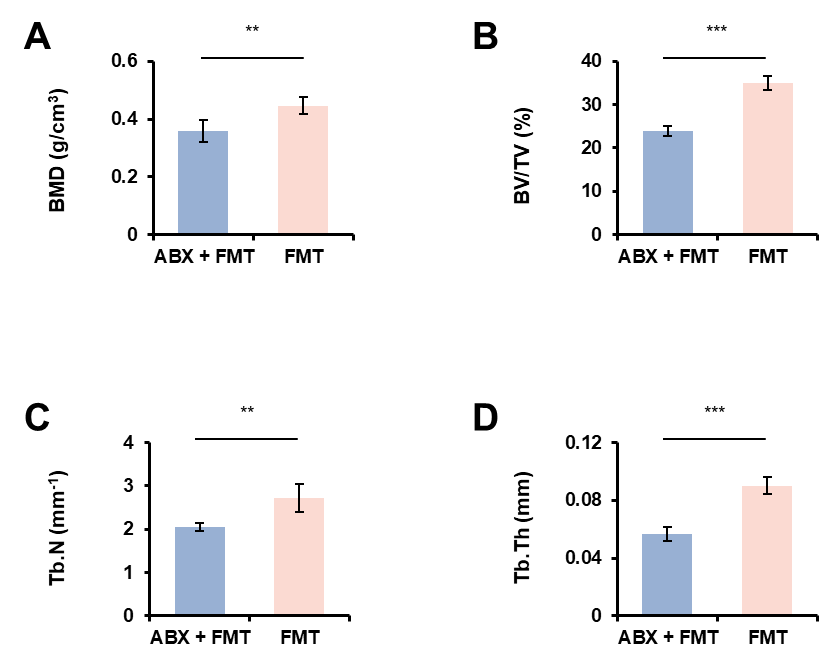


**Fig.S35.** Quantitative Micro-CT analysis of bone mass and microarchitecture (BMD, BV/TV, Tb.N, and Tb.Th) in the ABX + FMT and FMT groups. Data are presented as means ± SD. Statistical significance: **P* < 0.05, ***P* < 0.01, ****P* < 0.001.


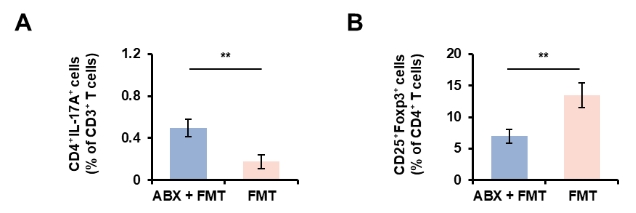


**Fig.S36.** Quantification of Th17 (A) and Treg (B) populations of different treatment groups. Data are presented as means ± SD. Statistical significance: **P* < 0.05, ***P* < 0.01, ****P* < 0.001.


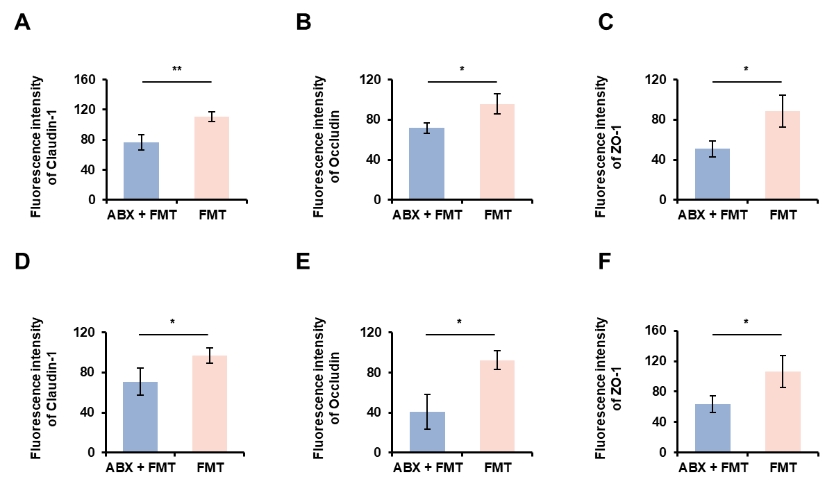


**Fig.S37.** Quantitative analysis of fluorescence intensity for Claudin-1 (A), Occludin (B), and ZO-1 (C) in ileum tissues, and for Claudin-1 (D), Occludin (E), and ZO-1 (F) in colonic tissues from ABX+FMT and FMT groups on day 49. Data are presented as means ± SD. Statistical significance: **P* < 0.05, ***P* < 0.01, ****P* < 0.001.


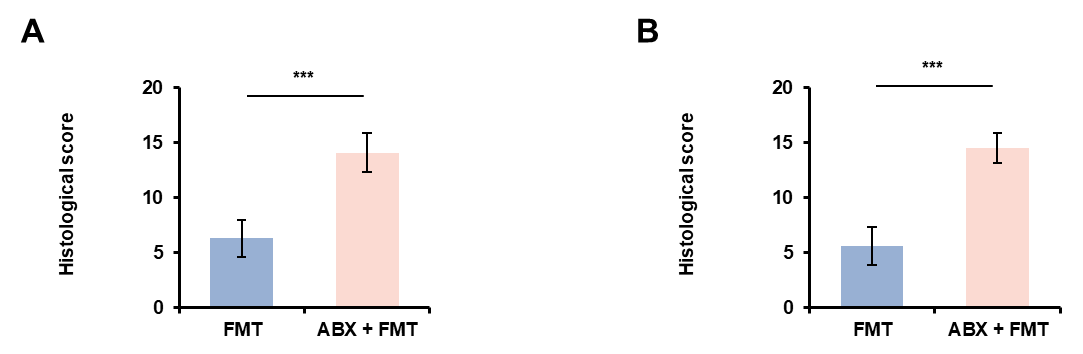


**Fig.S38.** Histological score of ileum (A) and colon (B) tissue from ABX+FMT and FMT groups. Data are presented as means ± SD. Statistical significance: **P* < 0.05, ***P* < 0.01, ****P* < 0.001.


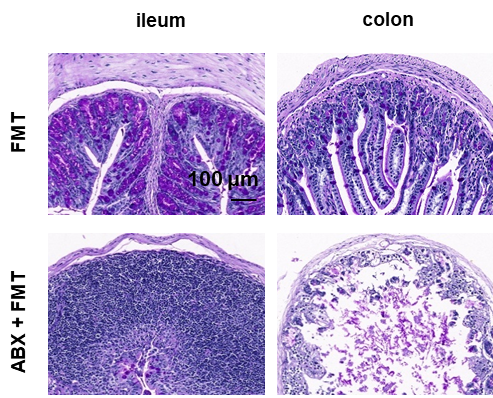


**Fig.S39.** PAS staining of ileum and colon tissues. Scale bars: 100 µm.


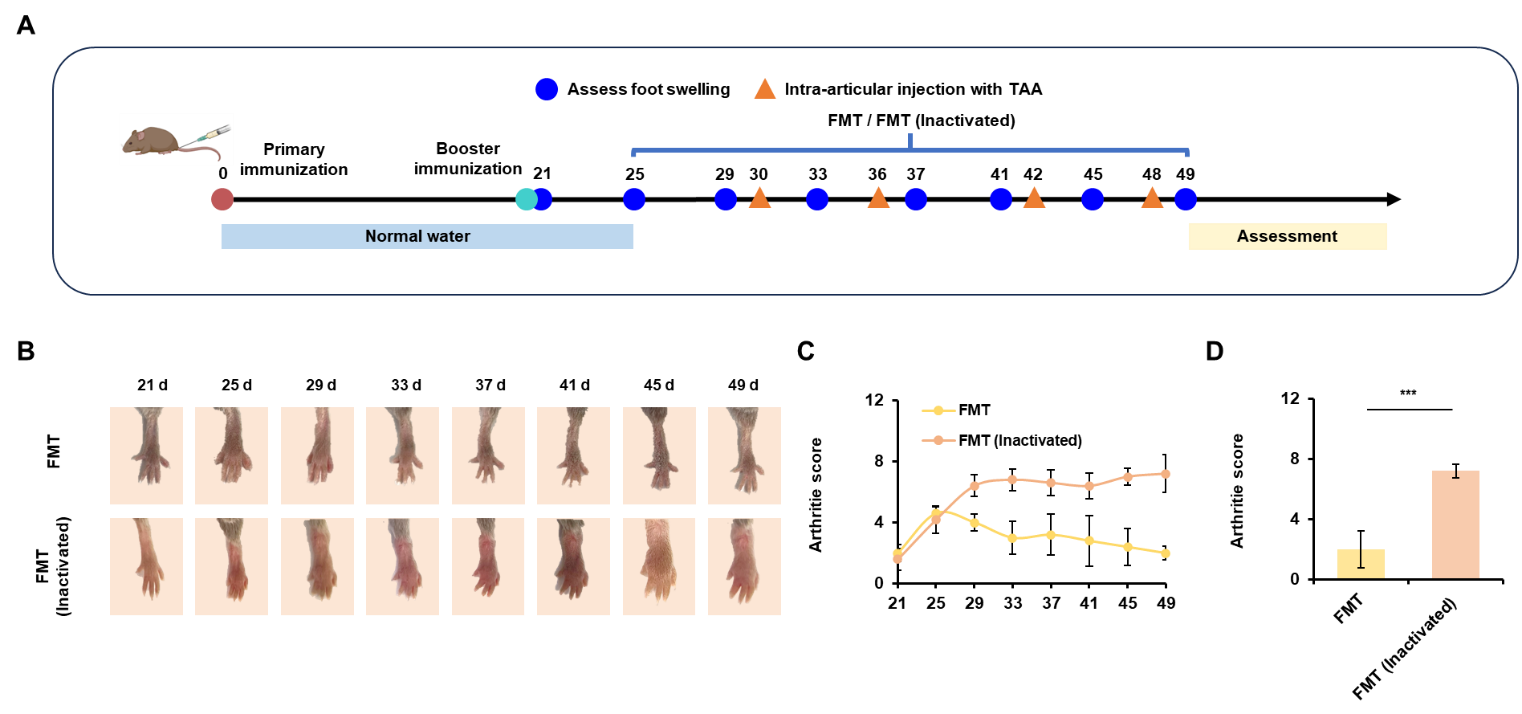


**Fig.S40.** Gut microbiota modulation by CG@GelMA is critical for enhancing the therapeutic efficacy of intra-articular TAA combination therapy. (A) Schematic overview of FMT and FMT（Inactivated）interventions in RA mice. (B) Representative joint morphology in different groups. (C–D) Arthritis scores recorded throughout the study (C) and on day 49 (D) in different groups. Data are presented as means ± SD. Statistical significance: **P* < 0.05, ***P* < 0.01, ****P* < 0.001.


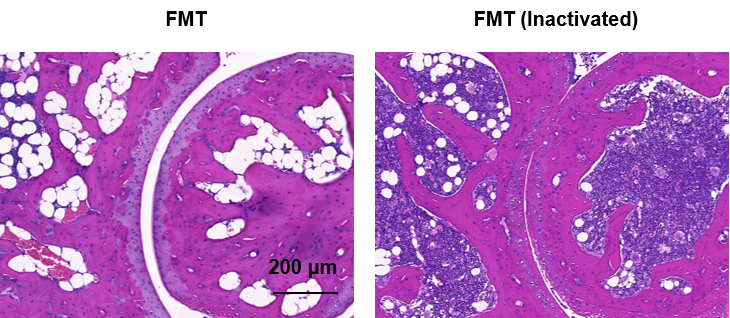


**Fig.S41.** HE staining of joint tissues in RA mice after FMT and inactivated FMT interventions at day 49. Scale bars: 200 µm.


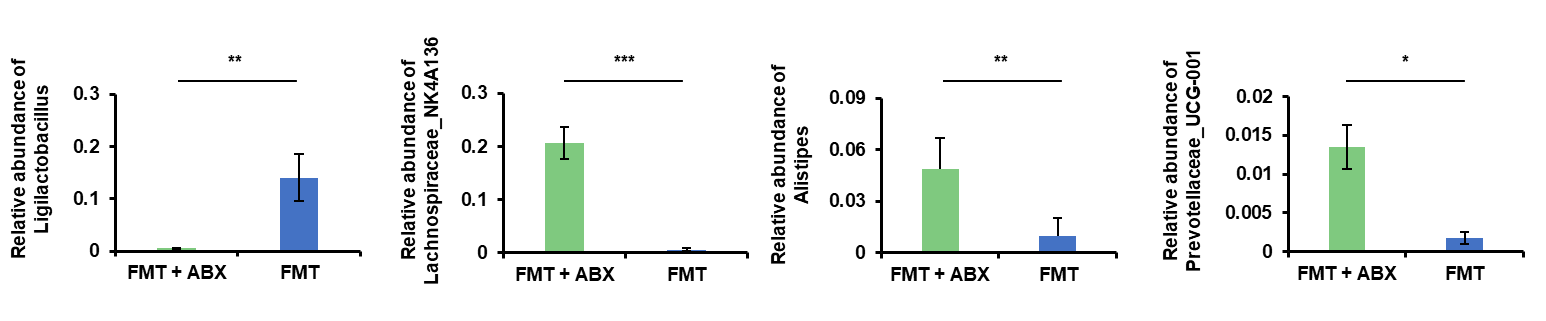


**Fig.S42.** Relative abundance of *Ligilactobacillus*, *Lachnospiraceae_NK4A136*, *Alistipes* and *Prevotellaceae_UCG-001.* Data are presented as means ± SD. Statistical significance: **P* < 0.05, ***P* < 0.01, ****P* < 0.001.


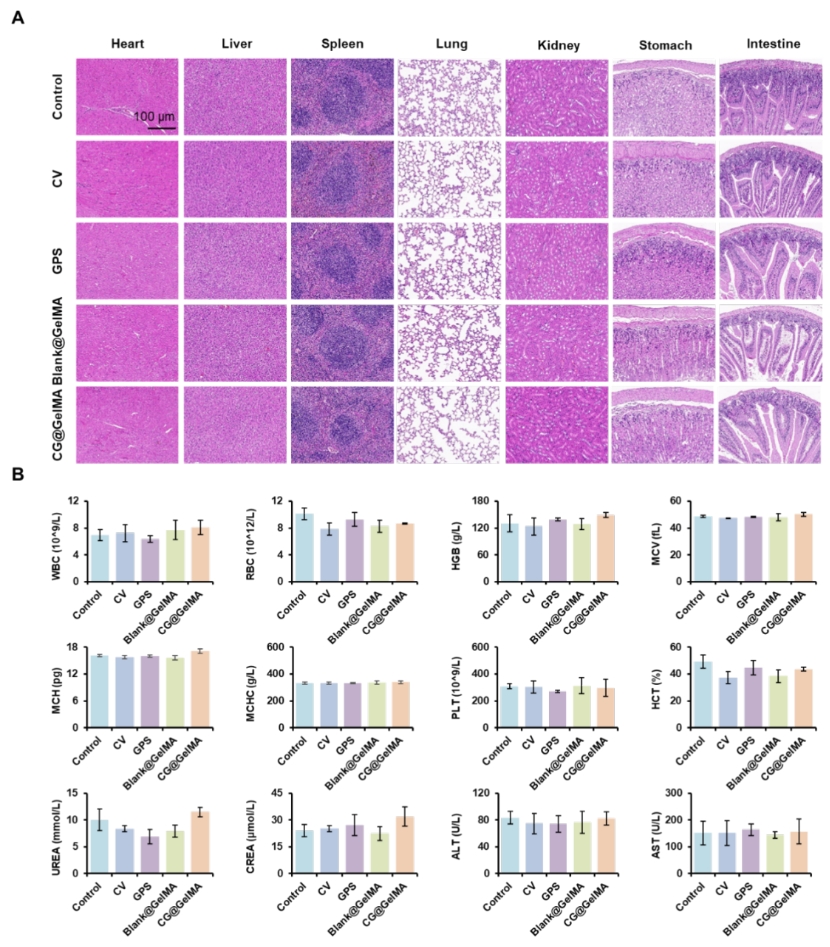


**Figure S43.** Safety evaluation after oral administration of various interventions.

(A) H&E staining of major organs—including heart, liver, spleen, lung, kidney, stomach, and intestine—from mice receiving different treatment.
(B) Blood routine and serum biochemical analyses performed after treatment. Data are presented as means ± SD.
